# Stroma-derived HGF drives metabolic adaptation of colorectal cancer to angiogenesis inhibitors

**DOI:** 10.18632/oncotarget.16942

**Published:** 2017-04-07

**Authors:** Alessia Mira, Virginia Morello, Maria Virtudes Céspedes, Timothy Perera, Paolo M. Comoglio, Ramon Mangues, Paolo Michieli

**Affiliations:** ^1^ Candiolo Cancer Institute, FPO, IRCCS, Candiolo, Turin, Italy; ^2^ Department of Oncology, University of Torino Medical School, Candiolo, Turin, Italy; ^3^ Biomedical Research Institute Sant Pau, Hospital de Sant Pau, Barcelona, Spain; ^4^ Centro de Investigación Biomédica en Red en Bioingeniería, Biomateriales y Nanomedicina, Barcelona, Spain; ^5^ OCTIMET Oncology Ltd., Oxford, United Kingdom

**Keywords:** colorectal cancer, HGF, anti-angiogenic therapy, resistance, tumor metabolism

## Abstract

The role of paracrine Hepatocyte Growth Factor (HGF) in the resistance to angiogenesis inhibitors (AIs) is hidden in xenograft models because mouse HGF fails to fully activate human MET. To uncover it, we compared the efficacy of AIs in wild-type and human HGF knock-in SCID mice bearing orthotopic human colorectal tumors. Species-specific HGF/MET signaling dramatically impaired the response to anti-angiogenic agents and boosted metastatic dissemination. In cell-based assays mimicking the consequences of anti-angiogenic therapy, colorectal cancer cells were completely resistant to hypoxia but extremely sensitive to nutrient deprivation. Starvation-induced apoptosis could be prevented by HGF, which promoted GLUT1-mediated glucose uptake, sustained glycolysis and activated autophagy. Pharmacological inhibition of GLUT1 in the presence of glucose killed tumor cells as effectively as glucose deprivation, and this effect was antagonized by HGF. Concomitant targeting of GLUT1 and HGF potently suppressed growth and dissemination of AI-resistant human tumors in human HGF knock-in SCID mice without exacerbating tumor hypoxia. These data suggest that stroma-derived HGF protects CRC cells against glucose starvation-induced apoptosis, promoting resistance to both AIs and anti-glycolytic agents. Combined inhibition of glucose metabolism and HGF/MET signaling (‘anti-METabolic therapy’) may represent a more effective CRC treatment compared to utterly blocking tumor blood supply.

## INTRODUCTION

Metastatic colorectal cancer (CRC) is one of the most common tumors in both men and women, and the second leading cause of cancer deaths worldwide [1]. The current approach to treating metastatic CRC favors the use of combination cytotoxic therapy (fluorouracil, leucovorin, oxaliplatin, capecitabine, irinotecan) plus biological agents targeting epidermal growth factor receptor (EGFR; cetuximab, panitumumab) [2]. More than half CRC patients, however, are not eligible to EGFR-targeted therapy because they bear *KRAS*- or *BRAF*-mutant tumors [3]. These patients, as well as those that have developed resistance to cetuximab or panitumumab, are typically addressed towards treatment with anti-angiogenic agents (bevacizumab, ziv-aflibercept, regorafenib) [4].

Angiogenesis inhibitors (AIs), and in particular bevacizumab, achieved encouraging results in advanced CRC [5, 6]. However, clinical testing of AIs in the adjuvant setting -where these kind of drugs are expected to be more effective based on their mechanism of action- was a major failure [7, 8]. Furthermore, most clinical trials testing agents targeting the vascular endothelial growth factor (VEGF) pathway in CRC provided evidence for increased progression-free survival but failed to demonstrate a substantial overall survival benefit, suggesting that anti-angiogenic treatment induces a biological adaptation leading to disease modification [9].

The biological mechanisms underlying resistance to AIs are not fully understood. Tumor hypoxia consequent to vessel pruning has been identified as a major cause of therapeutic failure and relapse [10]. It has been proven that a hypoxic micro-environment leads to clonal selection of tumor cells with increased invasive potential [11]. It has also been shown that hypoxia activates an invasive growth program orchestrated by hepatocyte growth factor (HGF) that leads cancer cells away from the hypoxic area, enabling them to escape anti-angiogenic therapy [12–14]. Finally, it has been demonstrated that AI-induced hypoxia cause tumor cells to rewire their glucose metabolism to fit the new micro-environment [15–17], although the molecular mechanisms and the cell-to-cell signals underlying this adaptation are still obscure.

HGF is a pleiotropic cytokine of mesenchymal origin that controls cell proliferation, motility, differentiation, and survival [18]. Its high affinity receptor, the MET tyrosine kinase, is frequently deregulated in human cancer [19]. HGF-driven MET activation plays a pivotal role in colorectal tumorigenesis and progression [20]. The vast majority of colorectal tumors express or overexpress the MET protein [21], and clinical findings indicate that activation of the MET pathway is a poor prognostic factor in CRC patients [22]. Although HGF/MET signaling typically controls epithelial-to-mesenchymal transition, a number of recent studies suggest that it may also be involved in the regulation of glucose metabolism [23–25].

In this study we hypothesized that HGF/MET signaling could be responsible not only for allowing CRC cells to physically escape anti-angiogenic therapy, but also for rewiring their metabolism in response to vessel pruning. To investigate this possibility, we exploited the inability of mouse HGF to fully activate human MET [26–28]. In fact, MET activation in colorectal tumors occurs primarily via paracrine HGF stimulation by the surrounding stroma [29]. When human cancer cells are transplanted into immuno-deficient mice, the growth of the developing tumor (expressing human MET) cannot be fully sustained by micro-environment-derived HGF (secreted by mouse stroma cells). Therefore, standard xenograft models do not represent an ideal system where to study the biological effects of paracrine HGF signaling.

We recently described the use of human HGF knock-in (hHGF KI) SCID mice for studying the role of stroma-derived HGF in the resistance to MET inhibitors [30]. In this mouse strain, the endogenous *HGF* gene has been replaced by homologous recombination with a cDNA encoding human HGF. Since human HGF activates both human and mouse MET, these animals are vital and fertile, and thanks to the SCID background, they support the growth of human tumor xenotransplants [30]. In contrast to transgenic mice that overexpress human HGF on top of mouse HGF [28], hHGF KI SCID mice express human HGF in place of mouse HGF, and since expression of the human gene is driven by the endogenous *HGF* gene promoter, hHGF KI SCID mice display fully physiological HGF expression levels [30].

To unmask the role of micro-environment-derived HGF in the response of CRC to AIs, we orthotopically injected human CRC cells bearing *KRAS* or *BRAF* mutations into wild-type (WT) and hHGF KI SCID mice, and compared the efficacy of different anti-angiogenic drugs in these two strains. To determine the effect of HGF on tumor metabolism, we compared the expression of glucose transporters in WT and hHGF KI SCID mice subjected to anti-angiogenic therapy. Finally, to dissect the mechanism by which HGF affects the cellular response to angiogenesis inhibition, we simulated the environmental conditions set by anti-angiogenic therapy in culture, and tested the impact of HGF on both classical biological parameters (proliferation, survival, motility) and cellular metabolic functions (glucose uptake, glycolysis, autophagy).

## RESULTS

### An orthotopic mouse model of colorectal cancer reproduces the tumor micro-environmental conditions and metastatic pattern found in cancer patients

To recapitulate the micro-environmental HGF/MET signaling conditions found in those oncological patients typically addressed towards anti-angiogenic therapy, we employed hHGF KI SCID mice (Suppl. Figure S1A) and MET-expressing human CRC cells bearing mutant *KRAS* (HCT-116) or *BRAF* (HT-29). HCT-116 and HT-29 cells, which displayed HGF-dependent MET activation (Suppl. Figure S1B), were engineered to express luciferase (luc) in order to track tumors and metastases. Micro-injection of HCT-116-luc and HT-29-luc cells into the cecum submucosa of hHGF KI SCID mice gave rise to orthotopic tumors within a few days, which could be monitored over time by bioluminescence analysis (Suppl. Figure S1C). Both HCT-116 and HT-29 tumors reproduced the pattern of metastatic dissemination observed in the clinic, which mainly involves the liver and the lungs. Metastases could be detected by histological examination and by bioluminescence analysis of explanted organs (Suppl. Figure S1D).

### Micro-environment-derived HGF promotes resistance to angiogenesis inhibitors

**Figure 1:**
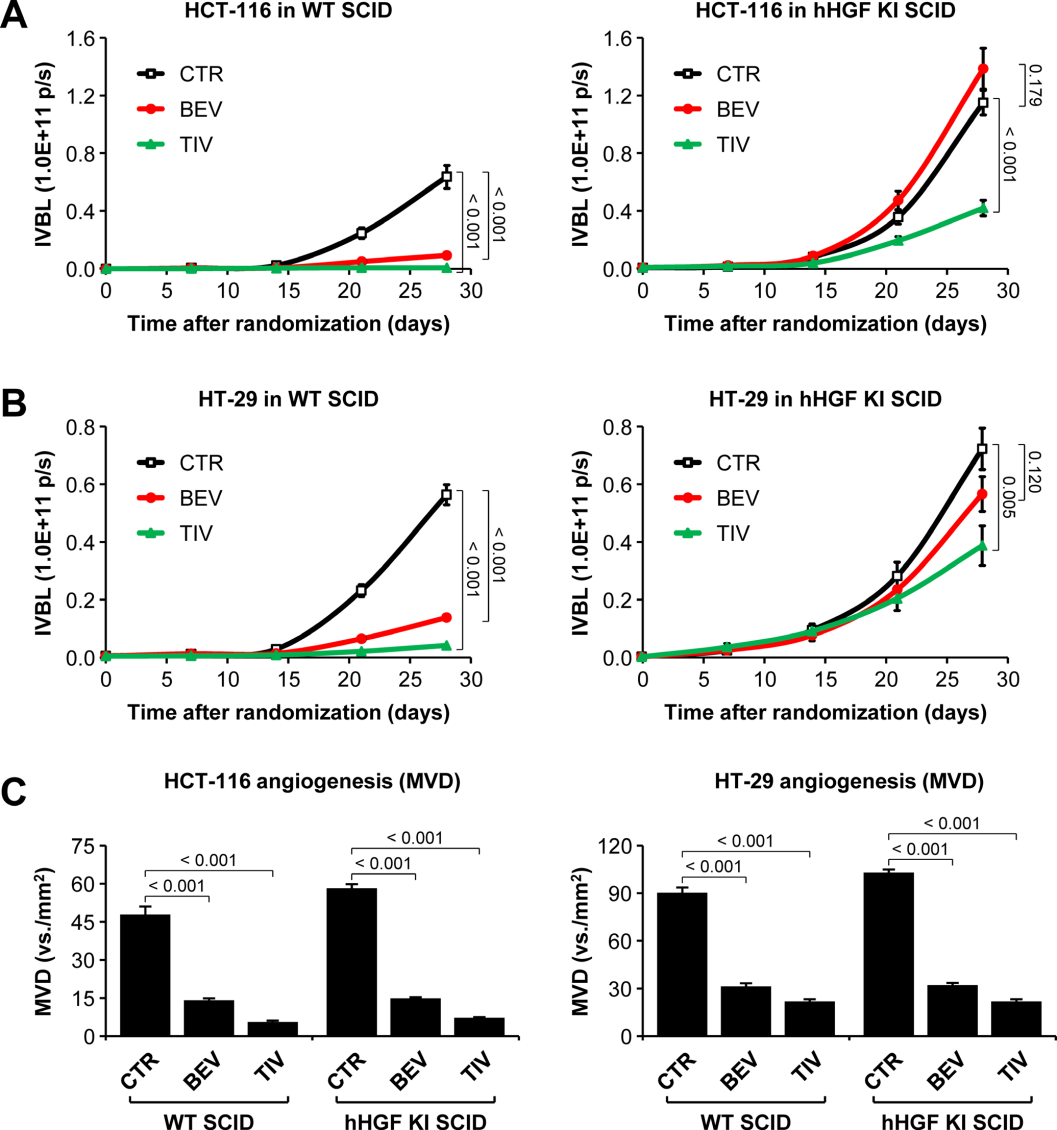
Micro-environment-derived HGF promotes resistance of orthotopic colorectal tumors to angiogenesis inhibitors **A**. HCT-116-luc cells were injected orthotopically into wild-type (WT) or human HGF knock-in (hHGF KI) SCID mice, and tumor-bearing mice were randomly assigned to 3 treatment arms: control (CTR), 15 mg/kg bevacizumab (BEV), and 10 mg/kg tivozanib (TIV). Tumor growth was followed over time by *in vivo* bioluminescence (IVBL). **B**. Same as in A but using HT-29-luc cells. **C**. Mean vessel density (MVD) of HCT-116 and HT-29 tumor tissues was determined by IHC using anti-CD146 antibodies. Values represent mean ± SEM. Statistical significance was calculated using a Student's T-test (A and B, *n* = 7; C, *n* = 21).

**Figure 2:**
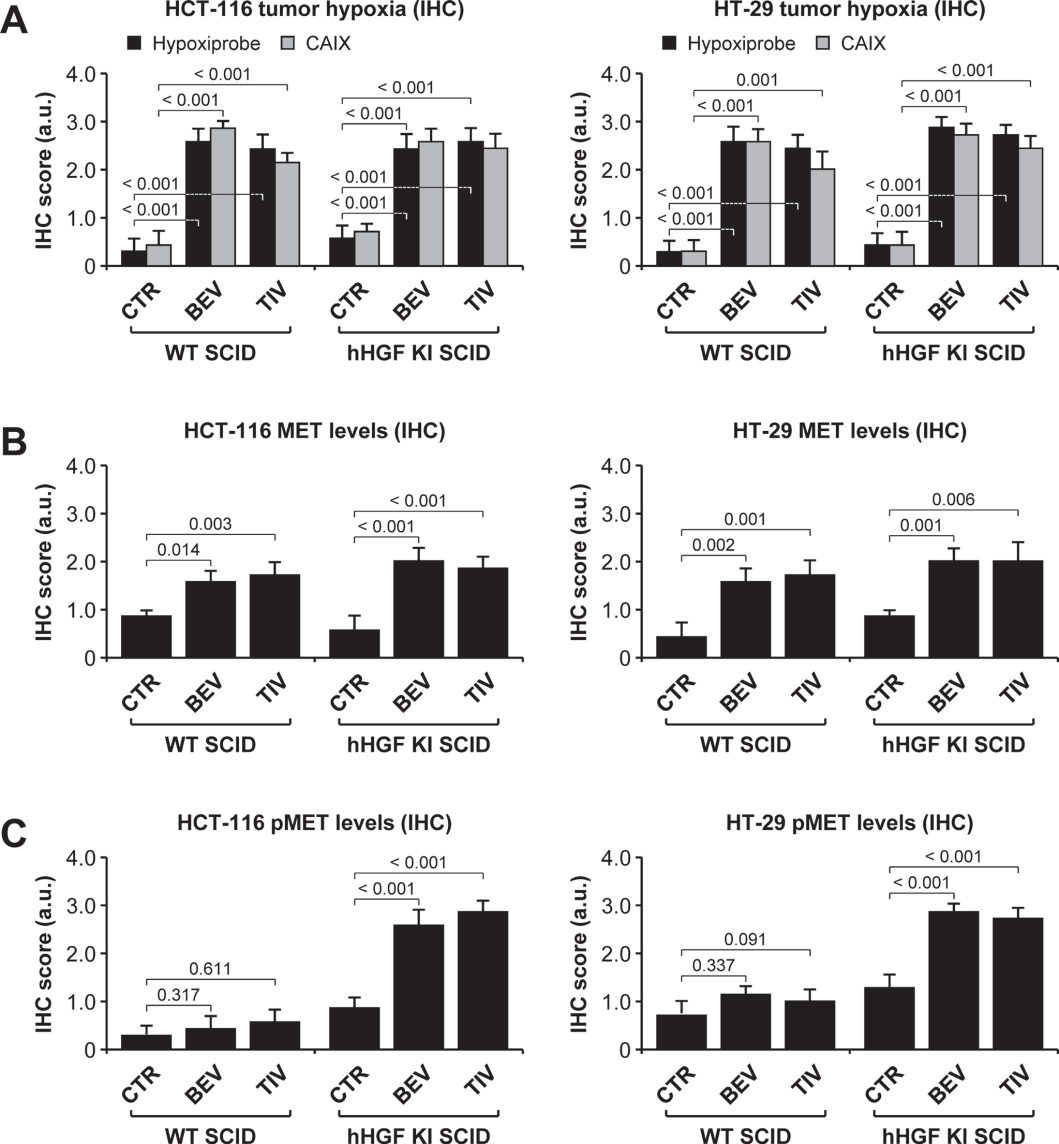
Anti-angiogenic therapy of orthotopic colorectal tumors promotes hypoxia-mediated, HGF-dependent MET activation **A**. Tumor hypoxia in HCT-116 and HT-29 tumors was determined by IHC using hypoxiprobe and anti-carbonic anhydrase IX (CAIX) antibodies. **B**. MET expression in HCT-116 and HT-29 tumors was determined by IHC analysis using anti-human MET antibodies. **C**. MET activation in HCT-116 and HT-29 tumors was determined by IHC analysis using anti-phospho MET antibodies. See Suppl. Figure S2 for representative images and score system description. Values represent mean ± SEM. Statistical significance was calculated using a Student's T-test (*n* = 7).

Human HCT-116-luc and HT-29-luc cells were orthotopically micro-injected into WT or hHGF KI SCID mice. Tumor-bearing animals were assigned to 3 treatment arms, which received no treatment, 15 mg/kg bevacizumab, or 10 mg/kg tivozanib (a pan-VEGF receptor small molecule inhibitor) [31]. Tumor growth and metastatic dissemination were assessed by *in vivo* and *ex vivo* imaging. Remarkably, both HCT-116 (Figure 1A) and HT-29 (Figure 1B) tumors grew significantly faster in hHGF KI SCID mice compared to WT SCID mice. Furthermore, while AIs markedly reduced tumor growth in WT SCID mice, they displayed a dramatically reduced therapeutic effect -if any- in hHGF KI SCID mice. Tumor micro-vessel density analysis revealed that impaired efficacy of AIs in hHGF KI SCID mice was not due to lack of anti-angiogenic activity (Figure 1C). In both tumor systems, angiogenesis inhibition by bevacizumab or tivozanib resulted in increased tumor hypoxia in either hHGF KI or WT SCID mice (Figure 2A), as determined by hypoxiprobe and carbonic anhydrase IX (CAIX) staining (see Suppl. Figure S2 for score system definition). Angiogenesis inhibition increased tumor hypoxia independently of species-specific HGF signaling (see Suppl. Figure S3 for representative images). Angiogenesis inhibition also increased MET expression in both hHGF KI and WT SCID animals (Figure 2B), but resulted in higher phospho-MET levels in hHGF KI SCID mice only (Figure 2C). These data suggest that species-specific HGF/MET signaling promotes resistance to AIs that is independent of the ability of the drugs to impair tumor angiogenesis.

### Anti-angiogenic therapy of orthotopic colorectal tumors promotes HGF-dependent metastatic dissemination

**Figure 3:**
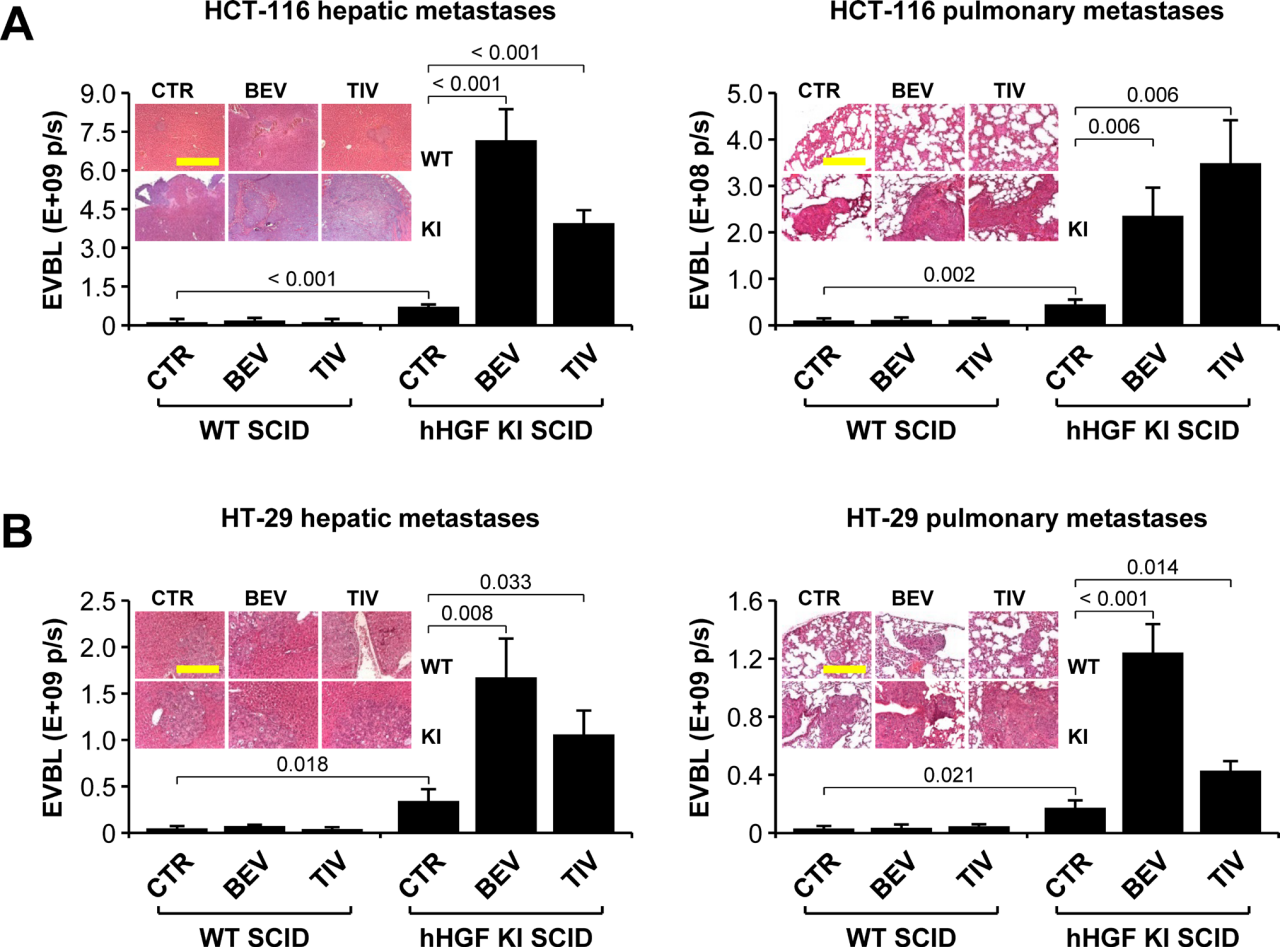
Anti-angiogenic therapy of orthotopic colorectal tumors promotes HGF-dependent metastatic dissemination **A**. Hepatic and pulmonary metastases in mice bearing HCT-116 tumors were determined by *ex vivo* bioluminescence (EVBL) analysis of explanted organs and by histological examination of tissue sections (inset). **B**. Same as in A but using mice bearing HT-29 tumors. Values represent mean ± SEM. Statistical significance was calculated using a Student's T-test (*n* = 7). Bar size: 250 μm.

Bioluminescence analysis of isolated organs revealed that metastatic dissemination was markedly HGF-dependent. In fact, livers and lungs extracted from hHGF KI SCID mice bearing HCT-116 (Figure 3A) or HT-29 (Figure 3B) tumors displayed dramatically higher bioluminescence levels compared to livers and lungs extracted from WT SCID animals. Autoptical and histological analysis confirmed this observation (see figure inset). Moreover, while anti-angiogenic therapy did not substantially affect liver or lung metastases in WT SCID mice, it strikingly enhanced both hepatic and pulmonary metastases in hHGF KI SCID mice, in both the HCT-116 (Figure 3A) and the HT-29 (Figure 3B) models. These data confirm the idea that CRC cells quickly adapt to anti-angiogenic therapy by engaging a HGF-dependent escape mechanism that leads them to disseminate into the host organism.

### HGF protects colorectal cancer cells against glucose starvation-induced apoptosis

**Figure 4:**
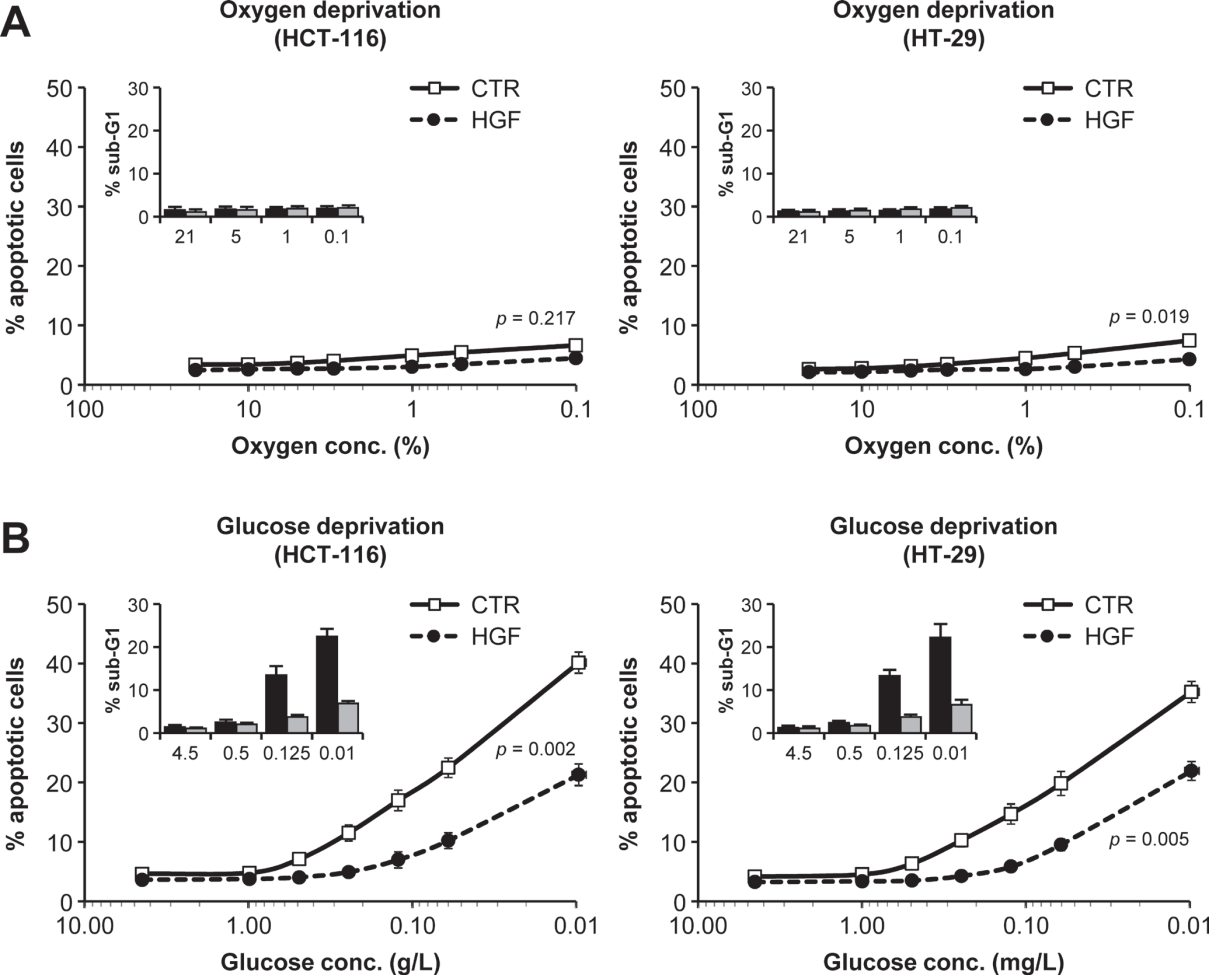
Oxygen and nutrient availability differentially affect colorectal cancer cell survival and proliferation **A**. HCT-116 and HT-29 cells were incubated in progressively lower oxygen concentrations in the absence or presence of HGF, and apoptosis was determined by the annexin/propidium iodide method. The percentage of cells in Sub-G1 (inset) was determined by DNA content analysis (see Suppl. Figure S3 for full cell cycle distribution data). **B**. HCT-116 and HT-29 cells were incubated in progressively lower glucose concentrations in the absence or presence of HGF. Apoptosis and cell cycle distribution was determined as in A. Values represent mean ± SEM. Statistical significance was calculated using a Student's T-test (*n* = 3).

By pruning tumor vessels, anti-angiogenic therapy increases tumor hypoxia and deprives cancer cells of nutrients, dramatically reducing glucose availability [32, 33]. In order to dissect the mechanism by which micro-environment-derived HGF protects CRC cells against anti-angiogenic treatment, we subjected HCT-116 and HT-29 cells to progressively lower concentrations of oxygen or glucose in the absence or presence of HGF. In both cell lines tested, oxygen deprivation did not cause apoptosis (Figure 4A) but rather G0/G1 arrest (Suppl. Figure S4A). In contrast, glucose deprivation dramatically increased the apoptotic rate (Figure 4B), causing a sub-G1 boost (see figure inset) and a striking S and G2/M drop (Suppl. Figure S4B). While HGF did not substantially affect cell viability in low oxygen, it contrasted hypoxia-induced cell cycle arrest, partially rescuing cell proliferation. Most remarkably, HGF protected CRC cells against glucose starvation-induced apoptosis, preventing the sub-G1 surge. We extended this analysis to a wider panel of MET-expressing human CRC cell lines with different genetic status (Suppl. Fig. S5). As shown in Table 1, cells bearing mutations in *KRAS* or *BRAF*, including HCT-116 and HT-29, were dramatically more sensitive to glucose deprivation compared to cells with WT *KRAS* and *BRAF*, and their survival in low glucose could be rescued by HGF. Oxygen deprivation had little effect on survival, regardless of genetic status. Altogether, these data suggest that *KRAS*- and *BRAF*-mutant CRC cells rely on glucose much more strictly than they depend on oxygen for survival, and that HGF extends the permissive range of glucose concentration.

**Table 1:** *KRAS*/*BRAF*-mutant CRC cells rely on glucose more strictly than they depend on oxygen for survival

			% apoptosis at 0.1% oxygen		% apoptosis at 0.1 g/L glucose	
CELL LINE	*KRAS*	*BRAF*	CTR	HGF	CTR	HGF
CACO-2	WT	WT	3.7 ± 0.1	2.5 ± 0.2	7.3 ± 0.4	4.2 ± 0.1
COLO-320-DM	WT	WT	3.3 ± 0.2	2.4 ± 0.2	6.5 ± 0.4	3.3 ± 0.2
LIM-1215	WT	WT	3.6 ± 0.3	2.4 ± 0.2	5.7 ± 0.4	4.1 ± 0.2
SW-48	WT	WT	4.6 ± 0.4	3.6 ± 0.2	6.5 ± 0.4	3.9 ± 0.2
DLD-1	G13D	WT	5.7 ± 0.4	4.3 ± 0.2	38.4 ± 1.3	20.6 ± 0.8
HCT-116	G13D	WT	6.7 ± 1.2	4.5 ± 0.9	40.4 ± 1.8	21.4 ± 1.8
LOVO	G13D	WT	6.0 ± 0.4	4.4 ± 0.4	33.6 ± 1.0	17.1 ± 0.7
LS-1034	A146T	WT	5.0 ± 0.4	3.0 ± 0.2	25.5 ± 1.7	12.5 ± 0.6
LS-123	G12S	WT	5.2 ± 0.6	2.8 ± 0.3	20.1 ± 1.0	11.0 ± 0.9
LS-174T	G12D	WT	6.1 ± 0.4	4.3 ± 0.4	32.4 ± 1.2	20.5 ± 0.6
LS-180	G12D	WT	6.6 ± 0.4	4.5 ± 0.3	26.1 ± 1.2	14.0 ± 0.7
SW-480	G12V	WT	5.9 ± 0.2	4.0 ± 0.1	24.0 ± 0.9	12.3 ± 0.9
HT-29	WT	V600E	7.5 ± 0.8	4.3 ± 0.3	22.9 ± 2.2	12.6 ± 1.3
RKO	WT	V600E	5.2 ± 0.3	3.0 ± 0.2	24.5 ± 0.6	12.1 ± 0.8
SW-1417	WT	V600E	4.4 ± 0.2	2.1 ± 0.2	24.2 ± 0.8	11.8 ± 0.9

A panel of human CRC cell lines with different *KRAS* and *BRAF* status was subjected to progressively lower concentrations of oxygen or glucose in the absence or presence of HGF, and the percentage of apoptotic cells was determined by flow cytometry using the annexin V/propidium iodide method. Values represent the mean ± SEM of 3 different measurements. CTR, control.

### HGF sustains colorectal cancer cell invasion in hypoxic conditions

**Figure 5:**
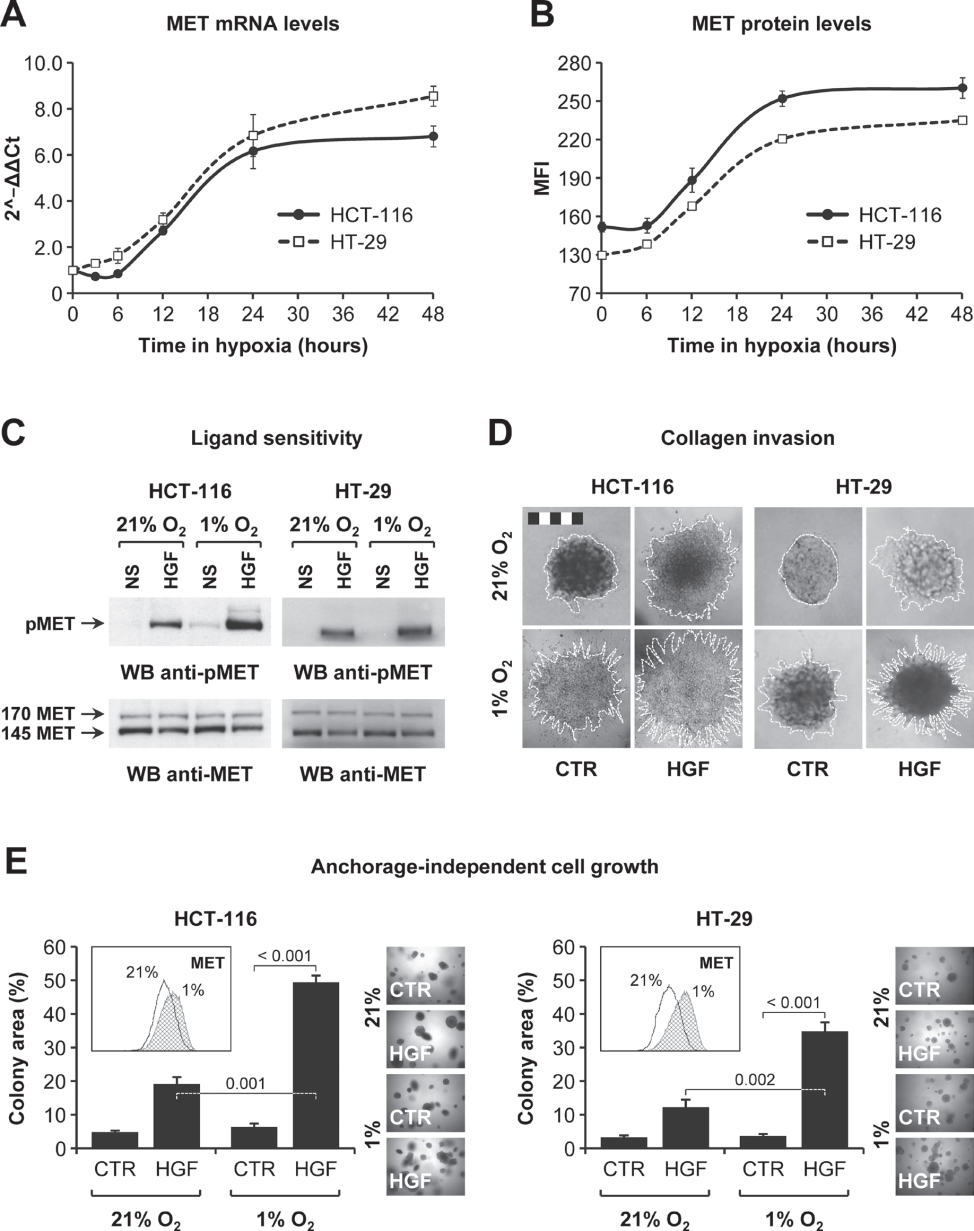
HGF sustains colorectal cancer cell invasion in hypoxic conditions **A**. MET mRNA levels in HCT-116 and HT-29 cells incubated in hypoxia (1% O_2_) were determined by RT-PCR. Data are expressed as 2^−∆ ∆ Ct^ compared to time zero. **B**. MET protein expression levels in HCT-116 and HT-29 cells incubated in hypoxia (1% O_2_) were determined by flow cytometry. MFI, mean fluorescence intensity. **C**. HCT-116 and HT-29 cells were pre-conditioned in 21% or 1% O_2_ and then stimulated with HGF. Phospho-MET (pMET) and total MET (MET) levels were determined by Western blotting (WB). NS, not stimulated. The upper 170 kDa MET band corresponds to the uncleaved, immature precursor. **D**. Collagen invasion by HCT-116 and HT-29 cells was determined in 21% or 1% O_2_ and in the absence or presence of HGF. Branching morphogenesis was analyzed by microscopy. Colony contour is highlighted with a white dotted line. Bar size: 500 μm. **E**. Anchorage-independent growth of HCT-116 and HT-29 cells pre-conditioned in 21% or 1% O_2_ was examined in a soft agar assay conducted in normoxia and in the absence or presence of HGF. Cells pre-conditioned in 1% O_2_ expressed higher levels of MET protein (see FACS inset). Values represent mean ± SEM. Statistical significance was calculated using a Student's T-test (*n* = 3).

Increased MET expression and HGF-dependent invasion under hypoxia has been described years ago and has been subsequently confirmed by other studies conducted in several model systems, including glioblastoma and pancreatic cancer [12–14]. To explore the effect of hypoxia in CRC models, we determined HCT-116 and HT-29 cell invasiveness in different oxygen atmospheres. In this set of experiments, hypoxia promoted MET mRNA expression (Figure 5A), increased MET protein levels exposed on the cell membrane (Figure 5B) and sensitized cells to HGF stimulation (Figure 5C). Moreover, hypoxia synergized with HGF in promoting branching morphogenesis (Figure 5D), selected for a more aggressive cell population that displayed higher MET levels and enhanced anchorage-independent cell growth in response to HGF (Figure 5E). These data indicate that hypoxia promotes HGF-dependent malignant progression of CRC cells, providing a mechanistic explanation for the higher metastatic levels observed in hHGF KI SCID mice treated with AIs.

### HGF promotes glucose uptake and sustains glycolysis by inducing GLUT1 expression

**Figure 6:**
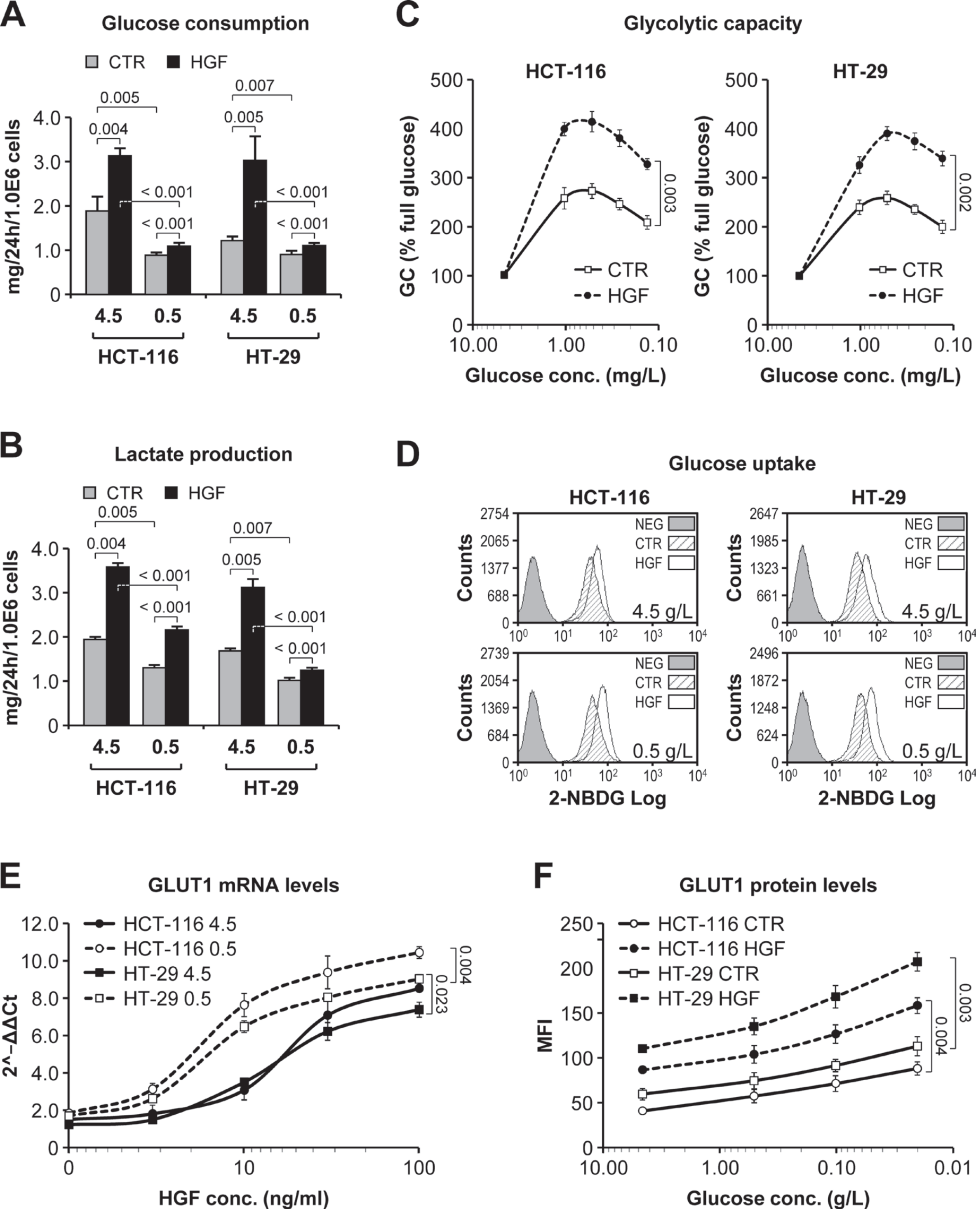
HGF sustains glycolysis in low glucose by promoting GLUT1-mediated glucose uptake **A**. Glucose consumption by HCT-116 and HT-29 cells grown in high (4.5g/L) or low (0.5g/L) glucose-containing medium and in the absence or presence of HGF was determined over a period of 24 hours. **B**. Same as in A but measuring lactate production. **C**. Glycolytic capacity (GC) of HCT-116 and HT-29 cells pre-incubated in progressively lower glucose concentrations and in the absence or presence of HGF was determined by Seahorse technology. GC is expressed as % relative to full glucose. **D**. Incorporation of the fluorescent glucose analogue 2-NBDG was determined by flow cytometry in HCT-116 and HT-29 cells pre-incubated in high (4.5g/L) or low (0.5g/L) glucose-containing medium and in the absence or presence of HGF. NEG, negative control. **E**. GLUT1 mRNA levels were determined by RT-PCR analysis of HCT-116 and HT-29 cells grown in high (4.5g/L) or low (0.5g/L) glucose-containing medium and in the presence of increasing HGF concentrations. Data are expressed as 2^−ΔΔCt^ compared to cells without HGF. **F**. GLUT1 protein levels were determined by flow cytometry analysis of HCT-116 and HT-29 cells grown in decreasing glucose concentrations and in the absence or presence of HGF. MFI, mean fluorescence intensity. Values represent mean ± SEM. Statistical significance was calculated using a Student's T-test (*n* = 3).

HGF increased glucose consumption by HCT-116 and HT-29 cells in both high (4.5 g/L) and low (0.5 g/L) glucose culturing conditions (Figure 6A), accompanied by higher lactate production (Figure 6B). To explore the role of HGF in regulating glycolysis, we incubated HCT-116 and HT-29 cells in decreasing glucose concentrations (4.5-0.125 g/L) in the absence or presence of HGF, and then measured glycolytic capacity in the presence of saturating glucose concentrations. Remarkably, HGF stimulation significantly enhanced the ability of CRC cells to perform glycolysis in limiting glucose concentrations (Figure 6C). To explore whether HGF directly affected glucose uptake, we cultured HCT-116 and HT-29 cells in high (4.5 g/L) or low (0.5 g/L) glucose concentrations in the absence or presence of HGF, and then incubated them with the fluorescent glucose analog 2-(N-(7-Nitrobenz-2-oxa-1,3-diazol-4-yl)Amino)-2-Deoxyglucose (2-NBDG). Glucose uptake was determined by flow cytometry. As shown in Figure 6D, HGF enhanced 2-NBDG uptake in both HCT-116 and HT-29 cells, and this effect was greater if cells were previously cultured in low glucose conditions. Consistent with this, HGF promoted the transcription of the *SLC2A1* gene (Figure 6E), resulting in higher GLUT1 protein levels (Figure 6F). Altogether, these data suggest that paracrine HGF signaling sustains CRC cell survival in limiting glucose concentrations by optimizing the efficiency of glucose influx and utilization.

### HGF potentiates colorectal cancer cell autophagic flux under glucose starvation

**Figure 7:**
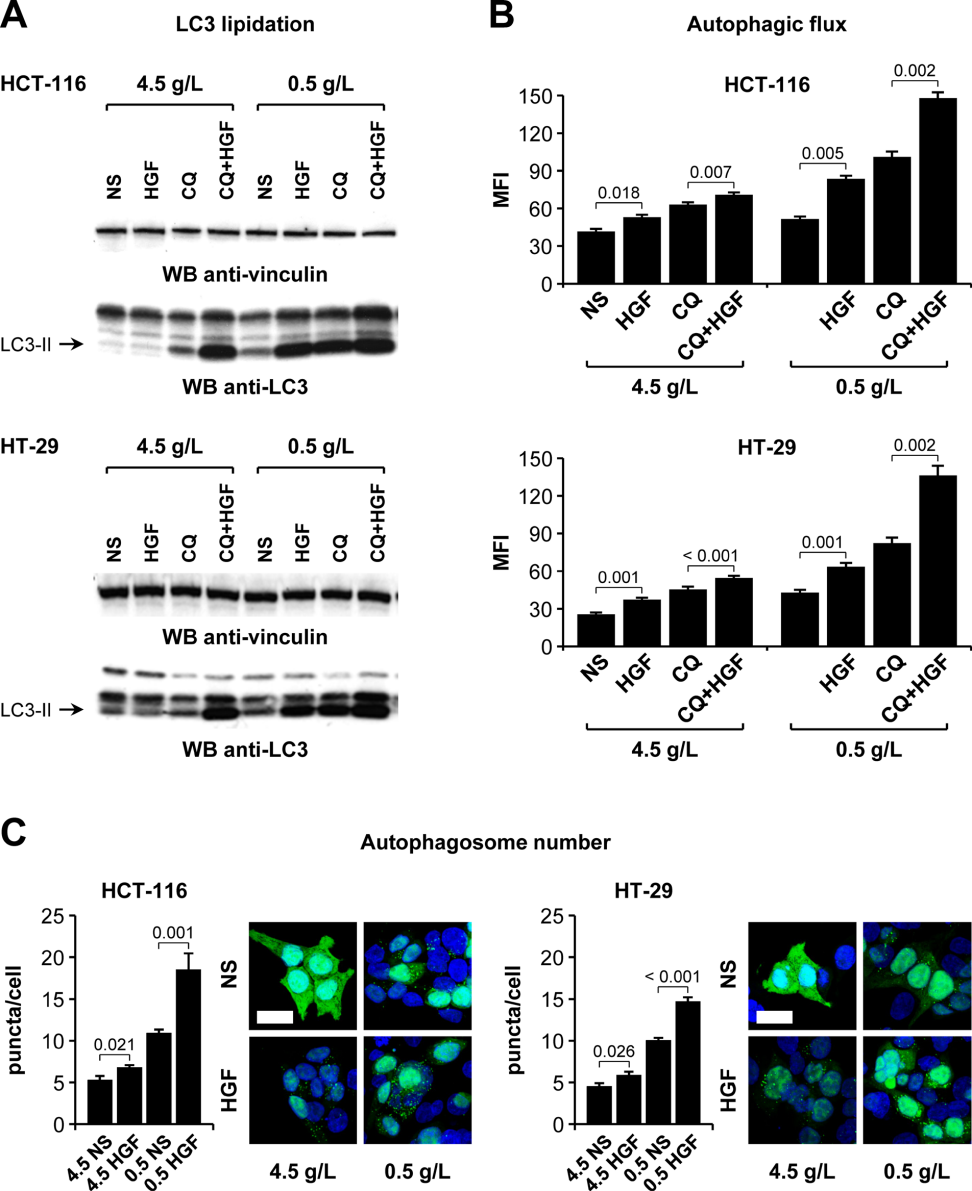
HGF potentiates colorectal cancer cell autophagic flux under glucose starvation **A**. HCT-116 and HT-29 cells were incubated in high (4.5 g/L) or low (0.5 g/L) glucose, in the absence or presence of HGF, and with or without chloroquine (CQ). LC3 lipidation was determined by Western blotting (WB) using anti-LC3 antibodies. The same blots were also analyzed using anti-vinculin antibodies. NS, not stimulated. The arrows indicate the lipidated LC3 isoform (LC3-II). **B**. HCT-116 and HT-29 cells were incubated as in A, permeabilized, and the cytosolic LC3 fraction was washed out. Autophagosome-associated LC3 levels were determined by flow cytometry. MFI, mean fluorescent intensity. **C**. HCT-116 and HT-29 cells ectopically expressing a GFP-LC3 fusion protein were cultured in high (4.5g/L) or low (0.5g/L) glucose-containing medium and in the absence or presence of HGF. The number of fluorescent GFP-LC3 puncta (corresponding to autophagosomes) was determined by confocal microscopy. Nuclei were stained with DAPI, and the number of fluorescent puncta was normalized on the number of DAPI-positive nuclei. Bar size: 20 μm. Values represent mean ± SEM. Statistical significance was calculated using a Student's T-test (B, *n* = 3; C, *n* = 10).

Growing evidence points at autophagy as one of the mechanisms that cancer cells adopt to escape the metabolically challenging conditions set by anti-angiogenic therapy [34]. To investigate a possible role of HGF in controlling autophagy, HCT-116 and HT-29 cells were incubated in high (4.5 g/L) or low (0.5 g/L) glucose, and in the absence or presence of HGF. Cells were also incubated with or without chloroquine, an inhibitor of lysosomal pH-dependent protein degradation. Changes in the autophagic flux were measured by Western blot analysis of microtubule-associated protein light chain 3 (LC3). In high glucose, HGF significantly increased LC3 lipidation in the presence of chloroquine (Figure 7A). In low glucose, a condition that typically induces an autophagic response, HGF increased LC3 lipidation per se and also enhanced chloroquine-induced LC3 lipidation. To obtain more quantitative data, we measured autophagosome-associated LC3 levels by flow cytometry. This analysis revealed that HGF enhanced autophagic flux much more markedly in low glucose compared to high glucose (Figure 7B). We also determined the number of autophagosomes under different culturing conditions. HCT-116 and HT-29 cells were transduced with a lentiviral vector encoding a GFP-LC3 fusion protein and then incubated in high or low glucose as above. The number of fluorescent puncta (corresponding to autophagosomes) per cell was determined by confocal microscopy. These experiments confirmed that HGF stimulation enhances autophagy more potently in cells that are grown in limiting glucose concentrations (Figure 7C). Altogether, these results suggest that paracrine HGF signaling increases CRC cell survival in glucose-poor conditions by activating autophagy, thereby contributing to maintain energy balance.

### Resistance to angiogenesis inhibitors in human HGF knock-in SCID mice is associated with HGF-dependent GLUT1 overexpression

**Figure 8:**
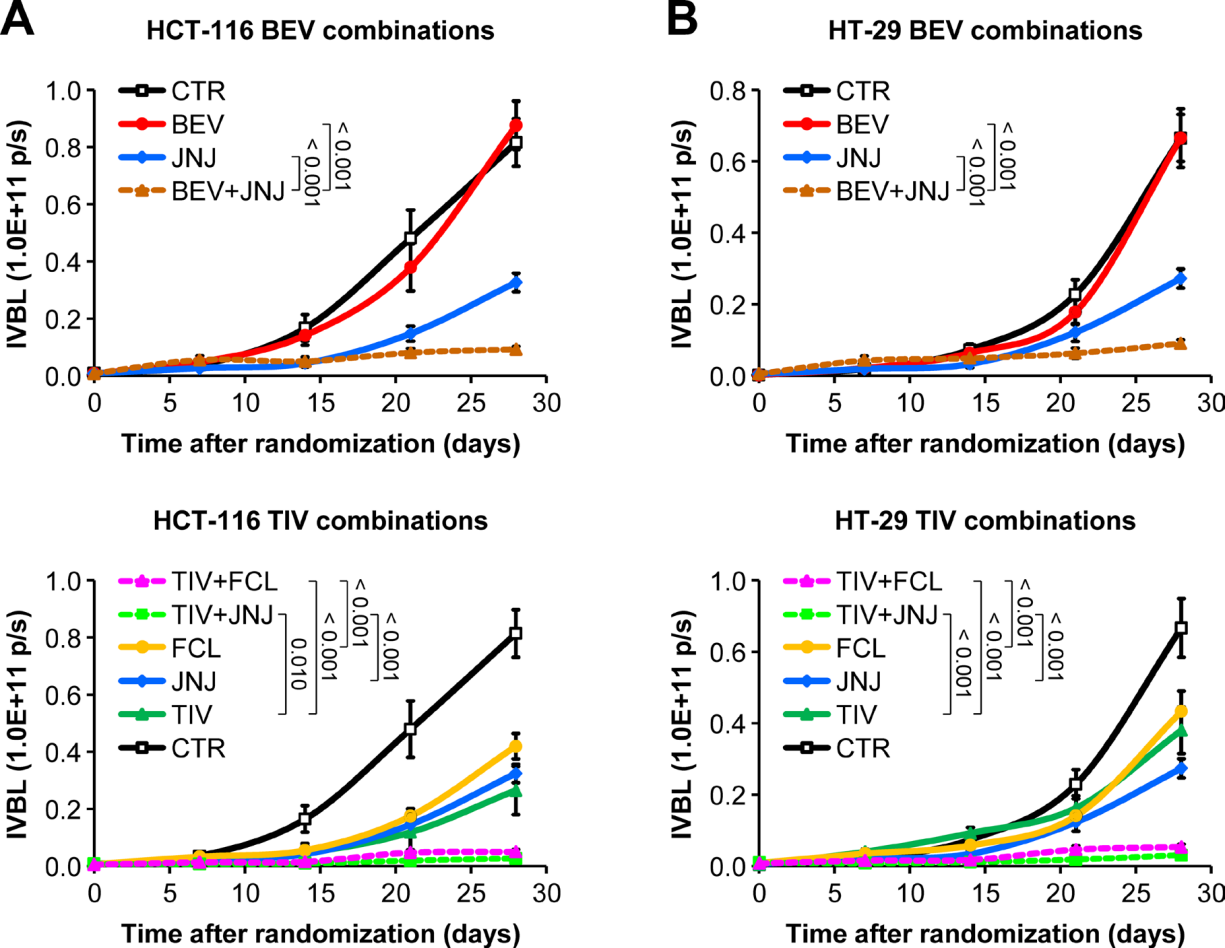
HGF/MET inhibition restores sensitivity to angiogenesis inhibitors in human HGF knock-in SCID mice **A**. HCT-116-luc cells were injected orthotopically into human HGF knock-in (hHGF KI) SCID mice, and tumor-bearing animals were randomly assigned to 8 treatment arms: control (CTR), 15 mg/kg bevacizumab (BEV), 10 mg/kg tivozanib (TIV), 40 mg/kg JNJ-38877605 (JNJ), 20 mg/kg ficlatuzumab (FCL), 15 mg/kg bevacizumab plus 40 mg/kg JNJ-38877605 (BEV+JNJ), 10 mg/kg tivozanib plus 40 mg/kg JNJ-38877605 (TIV+JNJ), and 10 mg/kg tivozanib plus 20 mg/kg ficlatuzumab (TIV+FCL). Tumor growth was followed over time by *in vivo* bioluminescence (IVBL). Results are shown in 2 different graphs for clarity. **B**. Same as in A but using HT-29-luc cells. Values represent mean ± SEM. Statistical significance was calculated using a Student's T-test (B, *n* = 7).

**Figure 9:**
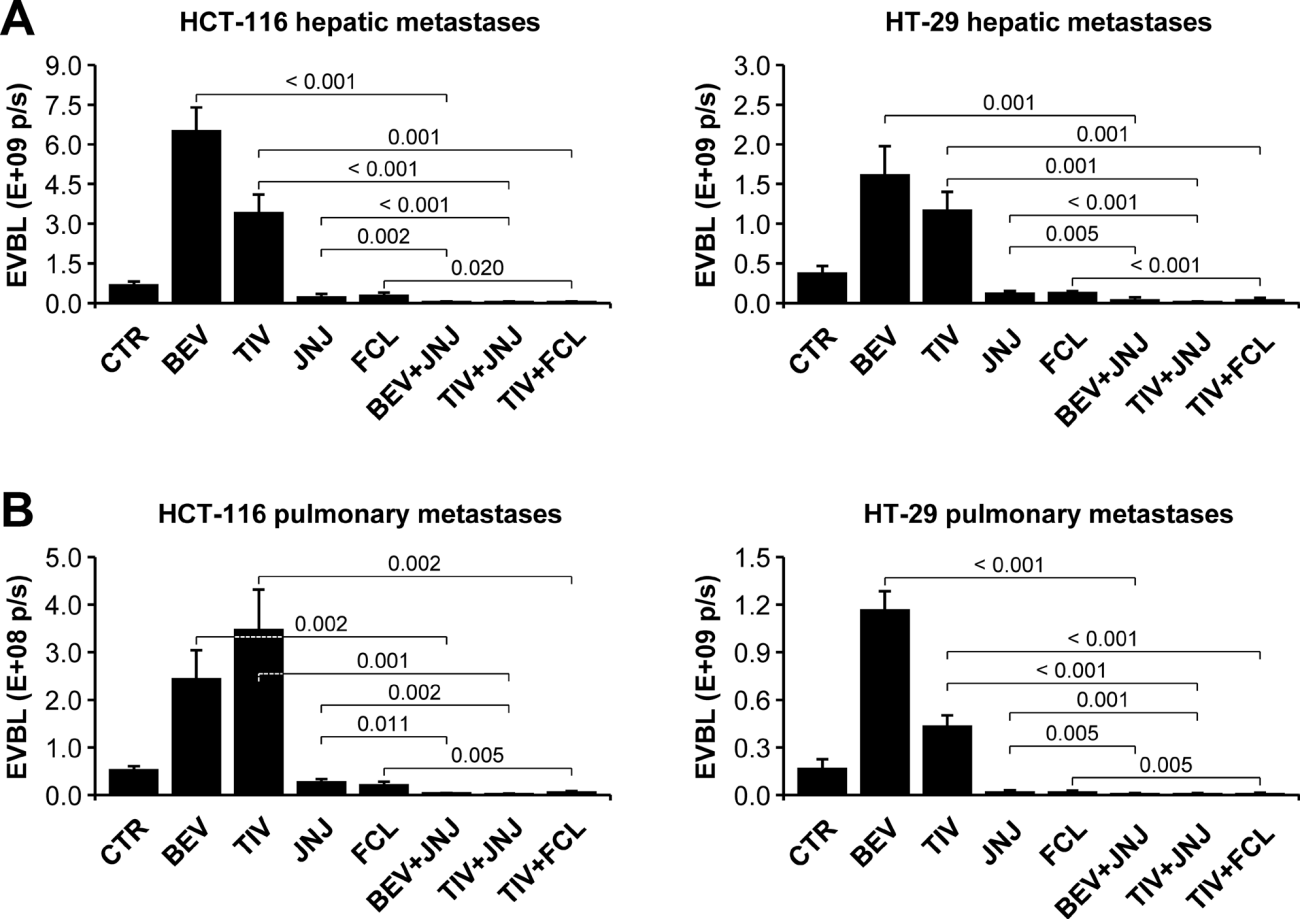
HGF/MET inhibition suppresses both spontaneous and angiogenesis inhibitor-induced metastatic spreading in human HGF knock-in SCID mice **A**. HCT-116-luc and HT-29-luc hepatic metastases were determined by *ex vivo* bioluminescence (EVBL) analysis of explanted livers. **B**. HCT-116-luc and HT-29-luc pulmonary metastases were determined by EVBL analysis of explanted lungs. Values represent mean ± SEM. Statistical significance was calculated using a Student's T-test (*n* = 7).

**Figure 10:**
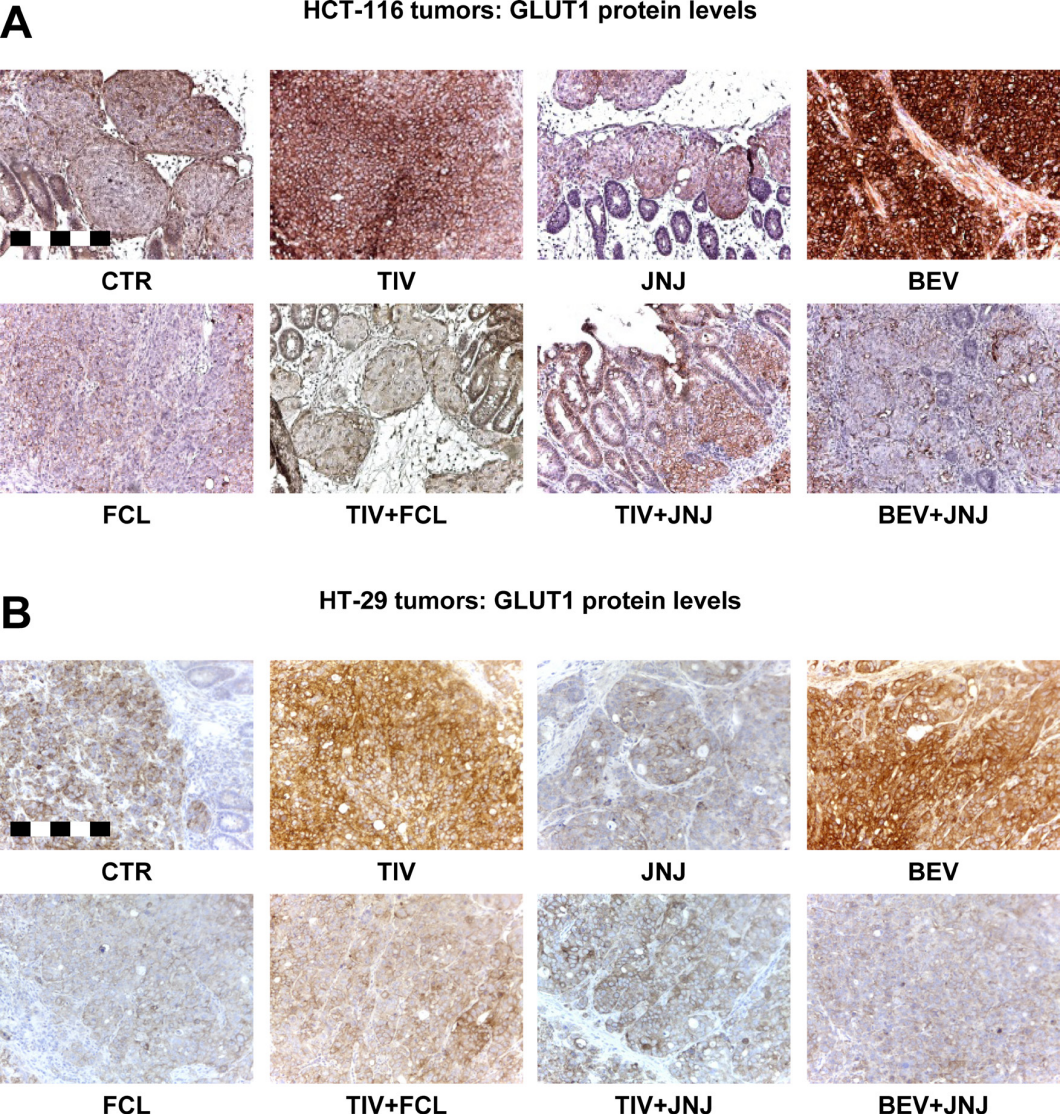
Resistance to angiogenesis inhibitors in human HGF knock-in SCID mice is associated with HGF-dependent GLUT1 overexpression **A**. GLUT1 protein expression was determined by staining sections form the HCT-116 tumors described in Figure 8A with a human GLUT1 antibody. Bar size: 250 μm. **B**. GLUT1 protein expression was determined by staining sections from the HT-29 tumors described in Figure 8B with a human GLUT1 antibody. Bar size: 250 μm.

The data presented so far suggest that HGF promotes a metabolic ‘salvage’ program allowing CRC cells to avoid an energetic crash when facing glucose shortage. We reasoned that pharmacological blockade of HGF/MET signaling could prevent this adaptive process and overcome acquired resistance to AIs. To test this hypothesis, we orthotopically micro-injected HCT-116-luc and HT-29-luc cells into hHGF KI SCID mice as described above, and then randomly assigned tumor-bearing animals to the following arms: no treatment; 15 mg/kg bevacizumab; 10 mg/kg tivozanib; 40 mg/kg JNJ-38877605 (a MET-selective small molecule tyrosine kinase inhibitor) [30]; 20 mg/kg ficlatuzumab (a HGF-neutralizing antibody) [35]; 15 mg/kg bevacizumab plus 40 mg/kg JNJ-38877605; 10 mg/kg tivozanib plus 40 mg/kg JNJ-38877605; and 10 mg/kg tivozanib plus 20 mg/kg ficlatuzumab. Treatment continued for 4 weeks. Remarkably, MET inhibition by JNJ-38877605 or HGF neutralization by ficlatuzumab completely restored sensitivity to anti-angiogenic therapy in both HCT-116 (Figure 8A) and HT-29 (Figure 8B) tumors. Moreover, although MET/HGF blockade did not prevent AI-induced hypoxia, it fully suppressed metastatic spreading of both HCT-116 and HT-29 CRC cells to either the liver (Figure 9A) or the lungs (Figure 9B). Interestingly, and consistent with our hypothesis that glycolytic rewiring is associated with resistance to AIs, anti-angiogenic therapy dramatically increased GLUT1 expression in both HCT-116 (Figure 10A) and HT-29 (Figure 10B) tumors, which could be fully reversed by HGF/MET inhibition. These results suggest that resistance of colorectal tumors to AIs is associated with HGF/MET-dependent GLUT1 overexpression.

### Pharmacological GLUT1 blockade impairs colorectal cancer cell survival which can be rescued by paracrine HGF signaling

**Figure 11:**
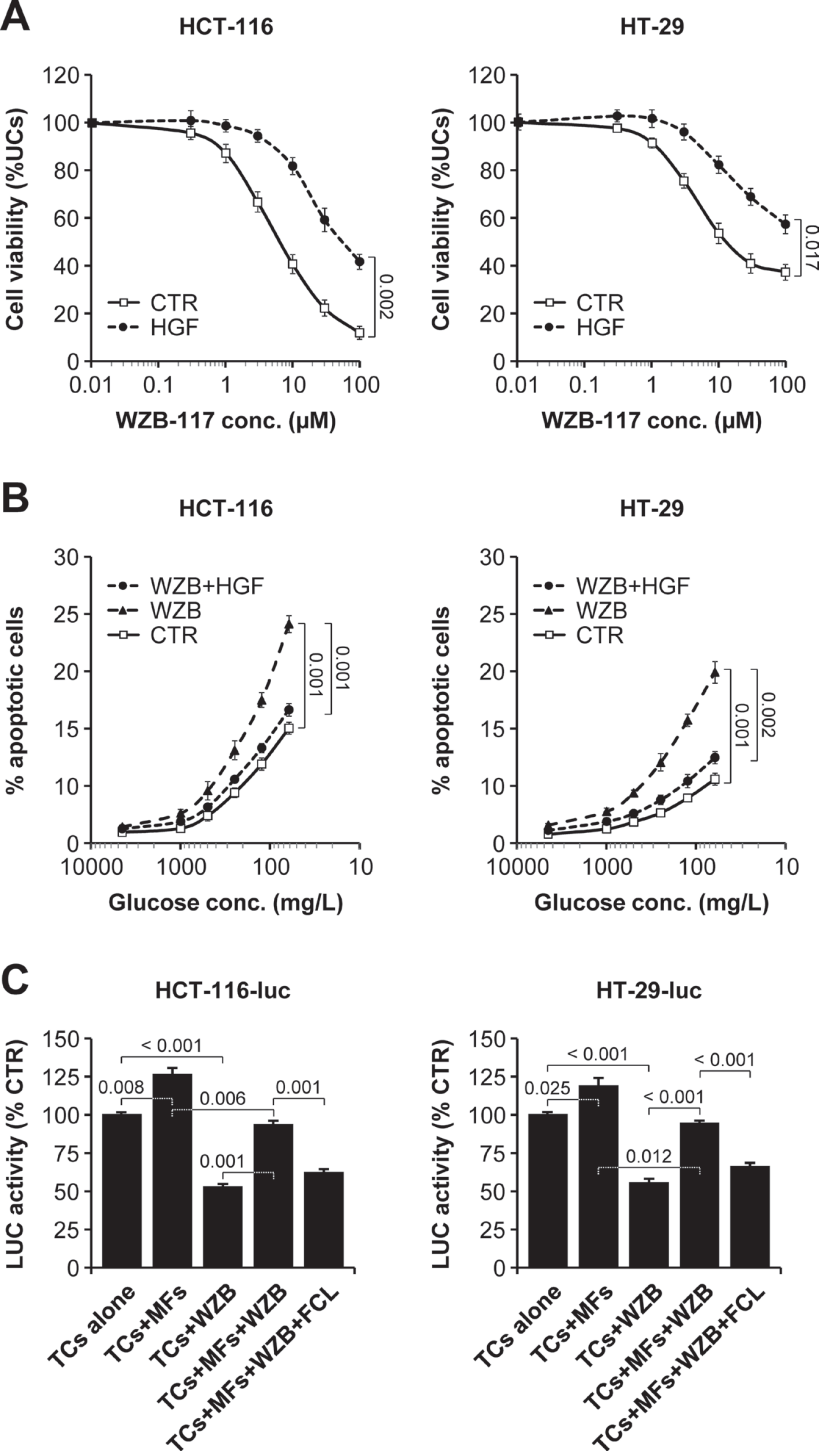
Pharmacological GLUT1 blockade impairs colorectal cancer cell survival which can be rescued by paracrine HGF signaling **A**. HCT-116 and HT-29 cells were cultured in the presence of increasing WZB-117 concentrations and in the absence or presence of HGF, and cell viability was determined by total ATP analysis. UCs, untreated cells. **B**. HCT-116 and HT-29 cells were incubated in decreasing concentrations of glucose, with 0 μmol/L or 20 μmol/L WZB-117 (WZB), and in the absence or presence of HGF. Apoptosis was determined by flow cytometry using the annexin/propidium iodide method. **C**. Luciferase-expressing tumor cells (TCs; HCT-116-luc or HT-29-luc) were seeded alone or together with human colon myofibroblasts (MFs) secreting approximately 1 μmol HGF/10^6^ cells/24 hours and then treated with 0 μmol/L or 20 μmol/L WZB-117 in the absence or presence of ficlatuzumab (FCL). Tumor cell viability was determined by measuring luciferase (LUC) activity. Values represent mean ± SEM. Statistical significance was calculated using a Student's T-test (*n* = 3).

HCT-116 and HT-29 cells were incubated with increasing concentrations (0.01-100 μmol/L) of the bis-hydroxybenzoate compound WZB-117, an irreversible blocker of GLUT1 [36], in the absence or presence of HGF. Cell viability was determined by total ATP analysis. As shown in Figure 11A, WZB-117 inhibited the growth of both HCT-116 and HT-29 cells in dose-dependent fashion, with an EC_50_ at 1.0 g/L glucose of 6.7 μmol/L and 11.2 μmol/L, respectively. HGF significantly protected HCT-116 and HT-29 cells against GLUT1 inhibition, increasing the EC_50_ by approximately one log (HCT-116, 53.7 μmol/L; HT-29, > 100 μmol/L). This analysis was extended to the same CRC cell panel used above for oxygen vs. glucose dependency. Consistent with their extraordinary vulnerability in low glucose conditions, *KRAS*- and *BRAF*-mutant CRC cells were significantly more sensitive to GLUT1 blockade compared to cells with WT *KRAS* and *BRAF* (Table 2). As seen for glucose deprivation, HGF antagonized the effect of WZB-117 and rescued cell survival in all cells analyzed. We next tested specifically whether HGF could protect CRC cells against WZB-117-induced apoptosis. HCT-116 and HT-29 cells were incubated in decreasing concentrations of glucose (4.5-0.06 g/L), with or without WZB-117, and in the presence or absence of HGF. Apoptosis was determined by flow cytometry using the annexin/propidium iodide method. As shown in Figure 11B, WZB-117 increased the apoptotic rate of both HCT-116 and HT-29 cells, and its pro-apoptotic effect was inversely proportional to glucose concentration. HGF stimulation significantly protected CRC cells against WZB-117-induced apoptosis at any glucose concentration. Finally, we tested the effects of WZB-117 and HGF in a stroma-tumor co-culture system using human colon myofibroblasts secreting approximately 1 μmol HGF/10^6^ cells/24 hours and luciferase-expressing CRC cells. HCT-116-luc or HT-29-luc cells were co-seeded with HGF-secreting myofibroblasts and then cultured with or without WZB-117 in the presence or absence of ficlatuzumab. Tumor cell viability was determined by measuring luciferase activity. As shown in Figure 11C, stromal cells protected CRC cells against WZB-111-induced apoptosis, and this effect could be neutralized by pharmacological inhibition of HGF.

**Table 2:** *KRAS/BRAF*-mutant CRC cells exhibit marked sensitivity to pharmacological GLUT1 blockade

			% cell viability at 20 μmol/L WZB-117	
CELL LINE	*KRAS*	*BRAF*	CTR	HGF
CACO-2	WT	WT	62.5 ± 2.8	74.3 ± 0.8
COLO-320-DM	WT	WT	58.2 ± 1.3	73.8 ± 1.2
LIM-1215	WT	WT	63.2 ± 1.4	74.4 ± 1.5
SW-48	WT	WT	63.1 ± 1.7	75.9 ± 1.7
DLD-1	G13D	WT	32.5 ± 1.5	65.0 ± 1.9
HCT-116	G13D	WT	28.7 ± 1.4	67.5 ± 2.5
LOVO	G13D	WT	27.3 ± 0.6	67.2 ± 1.0
LS-1034	A146T	WT	40.8 ± 0.9	61.9 ± 1.5
LS-123	G12S	WT	46.1 ± 0.9	67.3 ± 1.1
LS-174T	G12D	WT	34.0 ± 1.8	66.4 ± 1.4
LS-180	G12D	WT	34.2 ± 0.9	63.0 ± 1.5
SW-480	G12V	WT	35.6 ± 0.9	60.8 ± 1.1
HT-29	WT	V600E	44.0 ± 0.7	66.4 ± 1.5
RKO	WT	V600E	39.1 ± 0.7	70.6 ± 1.0
SW-1417	WT	V600E	42.1 ± 1.5	65.9 ± 0.8

A panel of human CRC cell lines with different *KRAS* and *BRAF* status was incubated with increasing concentrations (0.01-100 μmol/L) of WZB-117 in the absence or presence of HGF. Cell viability was determined by total ATP analysis. Values, expressed as % relative to untreated cells, represent the mean ± SEM of 3 different measurements. CTR, control.

### Concomitant inhibition of GLUT1 and HGF suppresses growth and dissemination of colorectal tumors resistant to anti-angiogenic inhibitors without exacerbating tumor hypoxia

**Figure 12:**
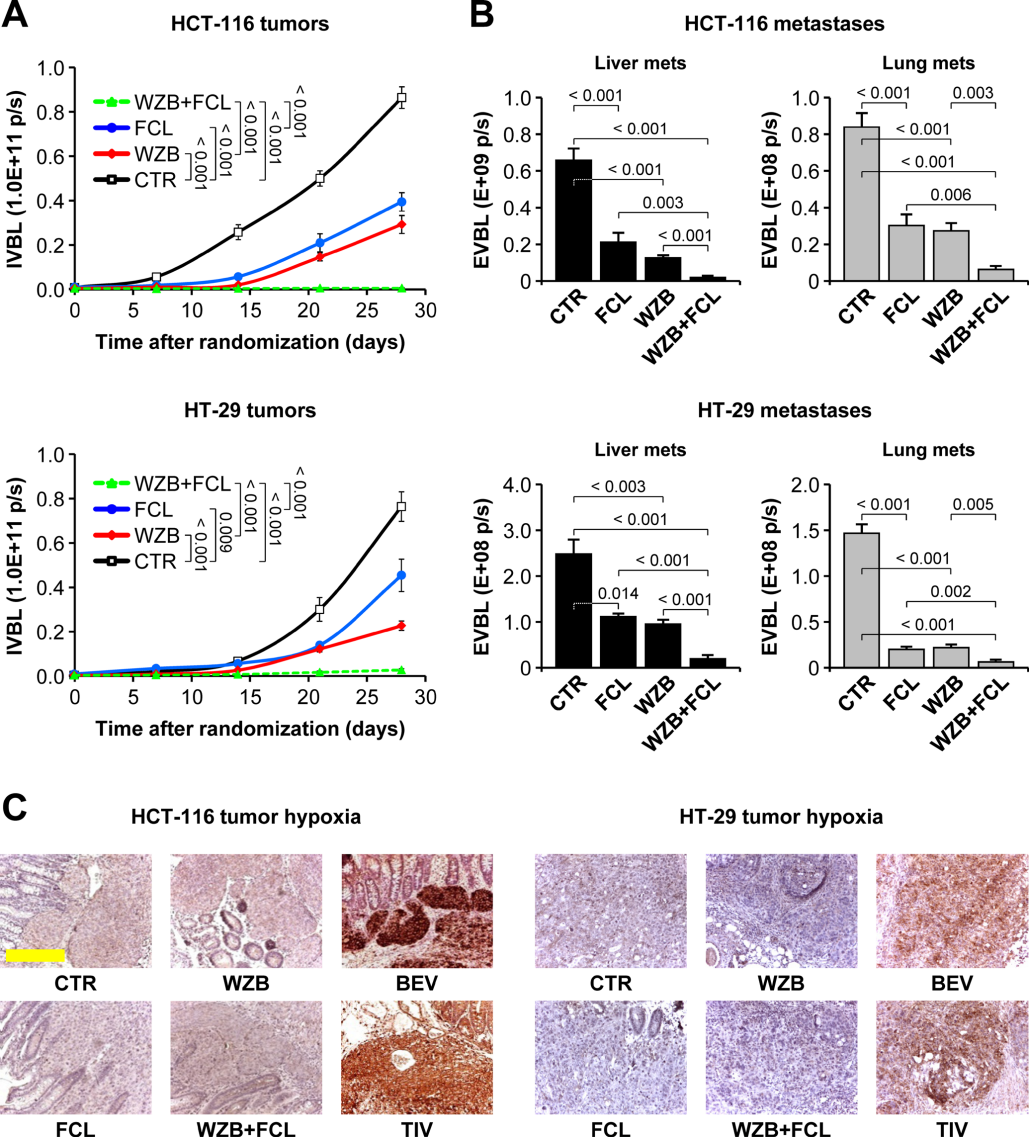
Concomitant inhibition of HGF/MET signaling and glucose uptake suppresses colorectal tumor growth and metastasis in human HGF knock-in mice **A**. HCT-116-luc and HT-29-luc cells were orthotopically injected into human HGF knock-in (hHGF KI) SCID mice, and tumor-bearing mice were randomly assigned to 4 treatment arms: control (CTR); 20 mg/kg ficlatuzumab (FCL); 10 mg/kg WZB-117 (WZB); 10 mg/kg WZB-117 plus 20 mg/kg ficlatuzumab (WZB+FCL). Tumor growth was followed over time by *in vivo* bioluminescence (IVBL). **B**. Hepatic and pulmonary metastases in mice bearing HCT-116 or HT-29 tumors were determined by *ex vivo* bioluminescence (EVBL) analysis of explanted organs. **C**. Tumor hypoxia was determined by IHC analysis of HCT-116 and HT-29 tumor sections stained with anti-carbonic anhydrase IX (CAIX) antibodies. Unlike utter vessel blockade by bevacizumab (BEV) and tivozanib (TIV), glucose uptake impairment by WZB-117 does not exacerbate tumor hypoxia. The BEV and TIV images come from the experiment described in Figures 1–3. Bar size: 250 μm. Values represent mean ± SEM. Statistical significance was calculated using a Student's T-test (*n* = 7).

The results presented so far provide experimental evidence that glucose withdrawal and not oxygen deprivation is crucial for killing *KRAS*/*BRAF*-mutant CRC cells, and that environmental HGF protects CRC cells against metabolic stress through GLUT1 upregulation. We therefore reasoned that selective targeting of GLUT1 would have been a cleaner and more effective therapeutic approach compared to generalized cutting of tumor blood supply, and that neutralization of HGF would have overcome micro-environment-mediated resistance to glucose uptake blockade. To test this hypothesis, we subjected hHGF KI SCID mice bearing orthotopic HCT-116-luc and HT-29-luc tumors to the following treatments: control; 20 mg/kg ficlatuzumab; 10 mg/kg WZB-117; 10 mg/kg WZB-117 plus 20 mg/kg ficlatuzumab. Tumor growth and metastatic dissemination was determined as described above. The results of this analysis provided relevant feedback on our work hypothesis. First, WZB-117 inhibited colorectal tumor growth much more effectively than AIs in the same conditions (Figure 12A vs. Figure 1A-1B). Second, in contrast to anti-angiogenic therapy, selective targeting of GLUT1 not only did not promote metastatic dissemination, but actually significantly inhibited it (Figure 12B vs. Figure 3A-3B). Third, neutralization of HGF by ficlatuzumab cooperated with metabolic blockade by WZB-117, inhibiting tumor growth and suppressing metastatic dissemination. Fourth and most important, and in striking contrast to AIs, GLUT1 inhibition did not exacerbate tumor hypoxia (Figure 12C vs. Figure 2A and Suppl. Figure S3).

## DISCUSSION

The strikingly different efficacy of AIs in WT and hHGF KI SCID mice suggests that stroma-derived HGF represents a major source of resistance to anti-angiogenic therapy of metastatic colorectal cancer, at least in the orthotopic models analyzed. Our cellular and metabolic analysis indicates that this phenomenon is explained by two major mechanisms, corresponding to distinct biological activities of HGF. On one hand, tumor hypoxia -exacerbated by anti-angiogenic drugs- sensitizes surviving CRC cells to paracrine HGF stimulation, which enhances tumor cell motility and invasion, thus allowing CRC cells to escape therapy and to form secondary colonies in distant organs. This mechanism confirms studies performed in other model systems [13, 14] and explains the higher incidence of metastasis observed in AI-treated hHGF KI SCID mice. On the other hand, stroma-derived HGF protects CRC cells against glucose starvation-induced apoptosis, allowing them to survive longer in a poorly vascularized tumor. The latter mechanism provides a molecular explanation for the inability of AIs to impair primary tumor growth in hHGF KI SCID mice and suggests that stroma-derived HGF controls tumor metabolism.

Our data show that HGF regulates the metabolic function of CRC cells in multiple ways. A first effect is transcriptional activation of the *SLC2A1* gene, resulting in increased expression of the GLUT1 protein. GLUT1 is the prototype of facilitated glucose transporters and is frequently overexpressed in tumors [37]. In CRC, expression of GLUT1 is strongly associated with disease progression and represents a validated predictive marker of poor prognosis [38, 39]. In rectal cancer patients, elevated GLUT1 expression is associated with high post-operative stage, presence of lymph node metastases and distal recurrence [40]. Higher GLUT1 levels may guarantee continuous glucose uptake, fueling glycolysis under low nutrient conditions. This possibility may represent a significant survival advantage for CRC cells when continuous glucose supply is compromised by anti-angiogenic therapy.

A second way by which HGF may influence CRC metabolism and contribute to maintain energy balance under starvation is through promotion of autophagy. Autophagy is an evolutionally conserved catabolic process by which cells destroy and recycle their macromolecules under metabolic stress in order to prevent an energetic crash [41]. HGF-mediated potentiation of autophagy may allow CRC cells to survive long enough until the ‘famine’ period is over, thus increasing the chances of escaping drug-induced nutrient deprivation and promoting resistance.

As for all the other tissues, the growth and survival of tumors depends on blood supply, which brings oxygen and nutrients. For some reason, however, most attention has been concentrated in the last two decades on the possibility to ‘suffocate’ the tumor (by reducing its oxygenation) rather than to ‘starve’ it (by hijacking its access to glucose). But is oxygen deprivation the real biological mechanism underlying the anti-tumor effect of AIs? Or on the contrary, is it a trigger for the malignant adaptation of cancer cells, leading to acquired resistance and disease progression?

The cell-based experiments aimed at dissecting the biological effects of anti-angiogenic therapy on CRC cells provide support for the latter hypothesis. In fact, all CRC cells tested, regardless of their genetic status, were relatively resistant to hypoxia, which had a mere cytostatic effect, but more sensitive to both glucose deprivation and GLUT1 blockade, which caused apoptosis. This dichotomy was particularly evident in *KRAS*/*BRAF*-mutant cells: while hypoxia increased their invasiveness and aggressiveness, prolonged hypoglycemia was completely incompatible with their survival. Altogether, these data point at nutrient rather than oxygen dependency as the real Achille's heel of *KRAS*- and *BRAF*-mutant tumors.

By pruning tumor blood vessels, AIs do deprive cancer cells of nutrients, thus in principle targeting this dependency, but at the same time cause tumor hypoxia, which represents a major stimulus for invasion and resistance. The data presented here suggest that, when inhibiting tumor angiogenesis, the benefits of reducing glucose availability are negatively compensated by the adverse effects of tumor hypoxia. In contrast, selective blockade of glucose metabolism achieves tumor starvation -which is what counts from a therapeutic viewpoint- without altering tumor oxygenation. Based on these results, it could be inferred that targeting glucose metabolism in CRC patients bearing *KRAS*- or *BRAF*-mutant tumors may be a safer and more effective strategy compared to utterly impairing tumor angiogenesis.

A variety of metabolism-targeting drugs are about to reach the clinic, including inhibitors of glucose uptake and utilization [42]. The availability of highly specific, clinical grade drugs will allow running dedicated clinical trials on CRC patients bearing *KRAS*- and *BRAF*-mutant tumors that compare the efficacy and safety of metabolism-targeting agents with that of angiogenesis inhibitors. Also, control of tumor metabolism by HGF may bring new life to the many HGF/MET inhibitors that are currently struggling to find an application in oncology. Possibly, the combined use of anti-metabolic agents and HGF/MET-targeted drugs (‘anti-METabolic therapy’) may be considered in order to unmask metabolic addictions and, most importantly, to prevent or delay the occurrence of acquired resistance.

## MATERIALS AND METHODS

### Cell culture

All tumor and stroma cell lines used in this study were purchased from the American Type Culture Collection (ATCC) and cultured as indicated by the provider. See online Suppl. Methods for more information on culturing conditions. Genetic identity of each cell line was confirmed by short tandem repeat profiling using Cell ID System (Promega).

### Orthotopic model of colorectal carcinoma

All animal protocols were approved by the Animal Research Ethical Committee, Fondazione Piemontese per la Ricerca sul Cancro - ONLUS, Candiolo, Italy, and by the Italian Ministry of Health. WT SCID CB17 SCID mice were purchased from Charles River. The hHGF KI SCID strain was obtained from AVEO Pharmaceuticals and has been described previously [30]. Orthotopic injection of HCT-116-luc or HT-29-luc cells was performed as described [43]. See online Suppl. Methods for a detailed protocol description and for more information on drug administration, *in vivo* and *ex vivo* bioluminescence analysis, and immunohistochemical examination of tumors.

### Oxygen *versus* glucose deprivation analysis

Cells were cultured in standard conditions until they reached 60% confluency, and then incubated in progressively lower concentrations of oxygen (21-0.1%) or glucose (4.5-0.01 g/L) in the absence or presence of 50 ng/ml human recombinant HGF (R&D Systems). After 48 hours, cells were processed for apoptosis and cell cycle analysis. For apoptosis analysis, cells were stained with a solution containing anti-annexin V antibodies and propidium iodide (eBioscience). The percentage of annexin V/propidium iodide-positive cells was determined by flow cytometry using a CyAn^TM^ ADP analyzer (Beckman Coulter). Cell cycle analysis was determined as described [44]. For hypoxia-induced invasion and RT-PCR analysis please refer to online Suppl. Methods.

### Metabolic analysis

Glucose and lactate concentration in the medium was determined using commercial kits (both from BioVision). For glucose uptake analysis, HCT-116 and HT-29 cells were pre-cultured in 4.5 g/L or 0.5 g/L glucose in the presence or absence of 50 ng/ml recombinant human HGF (R&D Systems) for 24 hours, and then incubated with 10 μmol/L 2-NBDG (Life Technologies) in glucose-free medium for 30 minutes. The 2-NBDG reaction was stopped by washing cells with pre-chilled PBS. Glucose uptake was quantified by measuring the fluorescent intensity of cells in the FL-1 channel using a CyAnTM ADP analyzer (Beckman Coulter). For glycolytic capacity analysis, the extracellular acidification rate (ECAR) was determined using a Seahorse XF96 analyzer (Seahorse Bioscience) as described [45]. See online Suppl. Methods for more details.

### Statistical analysis

Statistical significance was determined using a homoscedastic, two-tailed Student's t-test. A *p* value ≤ 0.05 was considered significant. In all Figures, values are expressed as mean of biological replicates ± SEM. Sample size is indicated in each Figure legend.

## SUPPLEMENTARY FIGURES



## Abbreviations

CRC: colorectal cancer
HGF: Hepatocyte Growth Factor
AI: angiogenesis inhibitor
EGFR: Epidermal Growth Factor Receptor
VEGF: Vascular Endothelial Growth Factor
WT: wild-type
KI: knock-in
SCID: severe combined immuno-deficiency syndrome
luc: luciferase
CAIX: carbonic anhydrase IX
CTR: control
BEV: bevacizumab
TIV: tivozanib
JNJ: JNJ-38877605
FCL: ficlatuzumab
IVBL: *in vivo* bioluminescence
MVD: mean vessel density
MFI: mean fluorecent intensity
