# Supplementary material for: Stroma-derived HGF drives metabolic adaptation of colorectal cancer to angiogenesis inhibitors
Source: Oncotarget. 2017 Apr 7;8(24):38193–213. doi: 10.18632/oncotarget.16942 (PMC5503526; doi:10.18632/oncotarget.16942)
Supplement: Supplementary file 1 [file oncotarget-08-38193-s001.pdf]

Stroma-derived HGF drives metabolic adaptation of colorectal cancer to angiogenesis inhibitors

Supplementary Material

METHODS

Cell culture

Immortalization of CCD-18Co cells was performed as described [1]. HGF secretion by CCD-18Co cells has been documented before [2] and was confirmed by ELISA using a human HGF Quantikine ELISA kit (R&D Systems). For experiments with defined concentrations of glucose, glucose-free DMEM medium (Gibco Life Sciences) was supplemented with D-glucose (Sigma-Aldrich) at the appropriate concentration. HCT-116-luc and HT-29-luc cells were generated by lentiviral vector transduction as described [1].

Orthotopic model of colorectal carcinoma

Orthotopic micro-injection of HCT-116 or HT-29 cells expressing luciferase was performed as described [3]. Briefly, HCT-116-luc or HT-29-luc cells (1.0×10^6 cells/mouse) were micro-injected into the cecum submucosa of six week-old WT or hHGF KI SCID mice using a glass micro-capillary. One week after, mice were injected with luciferin (Perkin Elmer) and their in vivo tumor bioluminescence was analyzed using an IVIS Lumina apparatus (Perkin Elmer). Animals were stratified into homogeneous groups based on bioluminescence signal and randomly assigned to the appropriate treatment arms ($n = 7$). Drugs were administered at the doses indicated in the text as follows: bevacizumab and ficlatuzumab, twice weekly by i.p. injection; tivozanib and JNJ-38877605, daily by oral gavage; WZB-117, daily by i.p. injection. Treatment continued for 4 weeks. Tumor growth was monitored weekly by IVIS analysis. At the end of the treatment, mice were injected with luciferin and pimonidazole HCl (hypoxiprobe), sacrificed, and their organs extracted for bioluminescence assessment. Following IVIS imaging, organs were formalin fixed, embedded in paraffin and processed for histological analysis.

Drugs

Tivozanib and ficlatuzumab were provided by AVEO Pharmaceuticals. JNJ-38877605 was supplied by Janssen Research & Development. Bevacizumab was obtained from our hospital pharmacy. WZB-117 was obtained from EMD Millipore and dissolved in ethanol.

Immuno-histochemical analysis

Upon autopsy, tumors and organs were explanted as described [1] and then formalin-fixed, paraffin-embedded and processed according to standard procedures. Tumor or tissue sections were analyzed by standard immuno-histochemistry protocols using the following antibodies: anti-CAIX rabbit polyclonal antibody (Novus Biologicals); anti-pimonidazole adducts mouse monoclonal antibody (hypoxiprobe); anti-MCAM CD146 rabbit monoclonal antibody (EMD Millipore); anti-phospho-MET rabbit polyclonal antibody (R&D Systems); anti-GLUT1 mouse monoclonal antibody (Novus Biologicals). Representative images were taken under a DMBL inverted microscope equipped with an ICC50 HD LCD camera (both from Leica). Vessel density was determined by microscopic analysis of tumor sections stained with anti-MCAM CD146 antibodies as described [4]. IHC staining of tumor hypoxia (both CAIX and hypoxiprobe), MET expression and MET activation was quantified using a score system based on the representative images shown in Suppl. Figure S2.

Hypoxia-induced invasion analysis

For MET auto-phosphorylation analysis, HCT-116 and HT-29 cells were cultured in normoxia (21% O_2) or hypoxia (1% O_2) in the presence of 1% FBS. After 24 hours, cells were stimulated with 50 ng/ml human recombinant HGF (R&D Systems)

for 10 minutes and then lysed as described [5]. MET auto-phosphorylation was assessed by Western blotting using anti-phospho-MET antibodies (Cell Signaling). Total MET levels were determined with anti-MET antibodies (Life Technologies). Cell surface MET expression was determined by flow cytometry using anti-MET antibodies (R&D Systems) and a CyAnTM ADP analyzer (Beckman Coulter). For branching morphogenesis assays, cells were seeded in a collagen layer as described [6] and then cultured for 48 hours in normoxia or hypoxia in the absence or presence of 50 ng/ml recombinant HGF (R&D Systems). Branching morphogenesis was evaluated by microscopy; representative images were taken for each group, and colony contour was highlighted manually. For anchorage-independent growth assays, colonies were stained with tetrazolium salts (Sigma Aldrich) and analyzed using a BD pathway HT 855 high-content imaging microscope (Becton Dickinson).

RT-PCR Analysis

For MET mRNA expression analysis in hypoxia, HCT-116 and HT-29 cells were incubated in 1% O_2 for the indicated times, and total RNA was extracted using Trizol (Life Technologies). Complementary DNA synthesis was achieved using the Reverse Transcription System (Promega) according to the manufacturer's instructions. RT-PCR was carried out by standard protocols using a Power Sybr PCR Master kit and a 7900 HT Abiprism Real-Time System (both from Applied Biosystems). Actin was chosen as a housekeeping gene for transcript normalization. The following primers were used:

ACTIN fw GGAGGAGCTGGAAGCAGCC
ACTIN rv GCTGTGCTACGTCGCCCTG
MET fw CCAAACAGGTGAAATATCTTGCA
MET rv TCAGCAACCTTGACTGTGAATTTT

For GLUT1 mRNA expression analysis, HCT-116 and HT-29 cells were grown in either high (4.5 g/L) or low (0.5 g/L) glucose culturing conditions for 24h in the absence or presence of 50 ng/ml recombinant HGF (R&D Systems), and total RNA was extracted using Trizol (Life Technologies). RT-PCR was carried out as above using the following primers:

GLUT1 fw TCACTGTGCTCCTGGTTCTG
GLUT1 rv CCTCGGGTGTCTTGTCACTT

Actin expression was used for normalization as described above.

Seahorse analysis

For glycolytic capacity analysis, the extracellular acidification rate (ECAR) was determined using a Seahorse XF96 analyzer (Seahorse Bioscience) as described [7]. Briefly, HCT-116 and HT-29 cells were seeded in Seahorse 96-well plates and cultured in the indicated glucose concentrations in the presence or absence of 50 ng/mL recombinant human HGF (R&D Systems). The assay cartridge was hydrated overnight at 37°C in a CO_2-free incubator. On the day of the assay, the growth medium was replaced with assay medium without glucose, pyruvate, serum and bicarbonate (Seahorse Biosciences), and incubated for 45 minutes at 37°C in a CO_2-free incubator. Glycolytic capacity was determined following sequential injections of D-glucose (10 mM), oligomycin (1 μM), and 2-deoxyglucose (100 mM) in accordance with Seahorse standard protocols. For each well, results were normalized to the protein content. Glycolytic capacity is defined as the proton production rate (PPR) after addition of oligomycin divided by the PPR before oligomycing addition.

Autophagy analysis

For assessing LC3 lipidation, HCT-116 and HT-29 cells were cultured in high (4.5 g/L) or low (0.5 g/L) glucose, with or without 10 μM cloroquine (Sigma-Aldrich), and in the presence or absence of 50 ng/mL HGF (R&D Systems). After 24 hours, half cells were lysed and analyzed by Western blotting using anti-LC3B (Novus Biologicals) and anti-vinculin (Sigma-Aldrich) antibodies. The remaining cells were permeabilized and stained using the FlowCellect Autophagy LC3 Antibody-based Assay Kit (EMD Millipore). Autophagosome-associated LC3 was determined using a CyAnTM ADP analyzer (Beckman Coulter). GFP-LC3 HCT-116 and HT-29 cells were generated by transduction with a LentiBrite GFP-LC3 lentiviral supernatant (EMD Millipore). Infected cells were sorted on the basis of their GFP expression using a MoFlo™ XDP cell sorter (Beckman Coulter). For confocal microscopy analysis, GFP-LC3 transduced cells were seeded on glass coverslips in 6-well plates and cultured in 4.5 g/L or 0.5 g/L glucose in the presence or absence of 50 ng/mL human recombinant HGF (R&D Systems) for 24 hours. Cells were then fixed in 4% para-formaldehyde and stained with DAPI (both from Sigma-Aldrich). Autophagosome number was determined by counting GFP-LC3 puncta on z-stacked images. For each condition, a minimum of 50 cells per replicate was counted. The number of GFP-LC3 puncta was normalized to the number of DAPI-positive nuclei.

GLUT1 inhibition assays

For viability assays, HCT-116 and HT-29 cells were seeded in 96-well plates (3,000 cells/well). The day after, cells were washed with PBS and further cultured in DMEM containing increasing concentrations of WZB-117 in the absence or presence of 50 ng/ml recombinant HGF (R&D Systems). After 48h, cell viability was determined using Cell Titer Glo (Promega) with a Victor X4 multilabel plate reader (Perkin Elmer). For apoptosis analysis, cells were seeded in 6-well plates ($3x10^5$ cells/well) and treated with 20 µM WZB-117 in the presence or absence of 50 ng/ml recombinant human HGF (R&D Systems). After 48 hours, cells were stained with a solution containing anti-annexin V antibodies and propidium iodide (eBioscience). The percentage of annexin V/propidium iodide-positive cells was determined by flow cytometry using a CyAnTM ADP analyzer (Beckman Coulter). For co-culturing experiments, HCT-116-luc and HT-29-luc cells and were seeded concomitantly with immortalized CCD-18Co myofibroblasts in 96-well plates (3,000 cells/well each cell type). The day after, cells were washed with PBS and then cultured in 4.5 g/L or 0.5 g/L glucose in the presence or absence of 60 µg/mL ficlatuzumab. Cell viability was determined 2 days later by measuring luciferase activity using a Luciferase Reporter Assay System kit (Promega). Samples were analyzed with a GloMax 96 Microplate Luminometer (Promega).

1. Basilico C, Hultberg A, Blanchetot C, de Jonge N, Festjens E, Hanssens V, Osepa SI, De Boeck G, Mira A, Cazzanti M, Morello V, Dreier T, Saunders M, et al. Four individually druggable MET hotspots mediate HGF-driven tumor progression. J Clin Invest. 2014; 124:3172-3186.
2. Vermeulen L, De Sousa E Melo F, van der Heijden M, Cameron K, de Jong JH, Borovski T, Tuynman JB, Todaro M, Merz C, Rodermond H, Sprick MR, Kemper K, Richel DJ, et al. Wnt activity defines colon cancer stem cells and is regulated by the microenvironment. Nat Cell Biol. 2010; 12:468-476.
3. Céspedes MV, Espina C, García-Cabezas MA, Trias M, Boluda A, Gómez del Pulgar MT, Sancho FJ, Nistal M, Lacal JC, Mangues R. Orthotopic microinjection of human colon cancer cells in nude mice induces tumor foci in all clinically relevant metastatic sites. Am J Pathol. 2007; 170:1077-1085.
4. Li W, Yang D, Wang S, Guo X, Lang R, Fan Y, Gu F, Zhang X, Niu Y, Yan X, Fu L. Increased expression of CD146 and microvessel density (MVD) in invasive micropapillary carcinoma of the breast: Comparative study with invasive ductal carcinoma-not otherwise specified. Pathol Res Pract. 2011; 12:739-746.
5. Basilico C, Pennacchietti S, Vigna E, Chiriaco C, Arena S, Bardelli A, Valdembri D, Serini G, Michieli P. Tivantinib (ARQ197) displays cytotoxic activity that is independent of its ability to bind MET. Clin Cancer Res. 2013; 19:2381-2392.
6. Medico E, Mongiovi AM, Huff J, Jelinek MA, Follenzi A, Gaudino G, Parsons JT, Comoglio PM. The tyrosine kinase receptors Ron and Sea control "scattering" and morphogenesis of liver progenitor cells in vitro. Mol Biol Cell. 1996; 4:495-504.
7. Dalva-Aydemir S, Bajpai R, Martinez M, Adekola KU, Kandela I, Wei C, Singhal S, Koblinski JE, Raje NS, Rosen ST, Shanmugam M. Targeting the metabolic plasticity of multiple myeloma with FDA-approved ritonavir and metformin. Clin Cancer Res. 2015; 5:1161-1171.

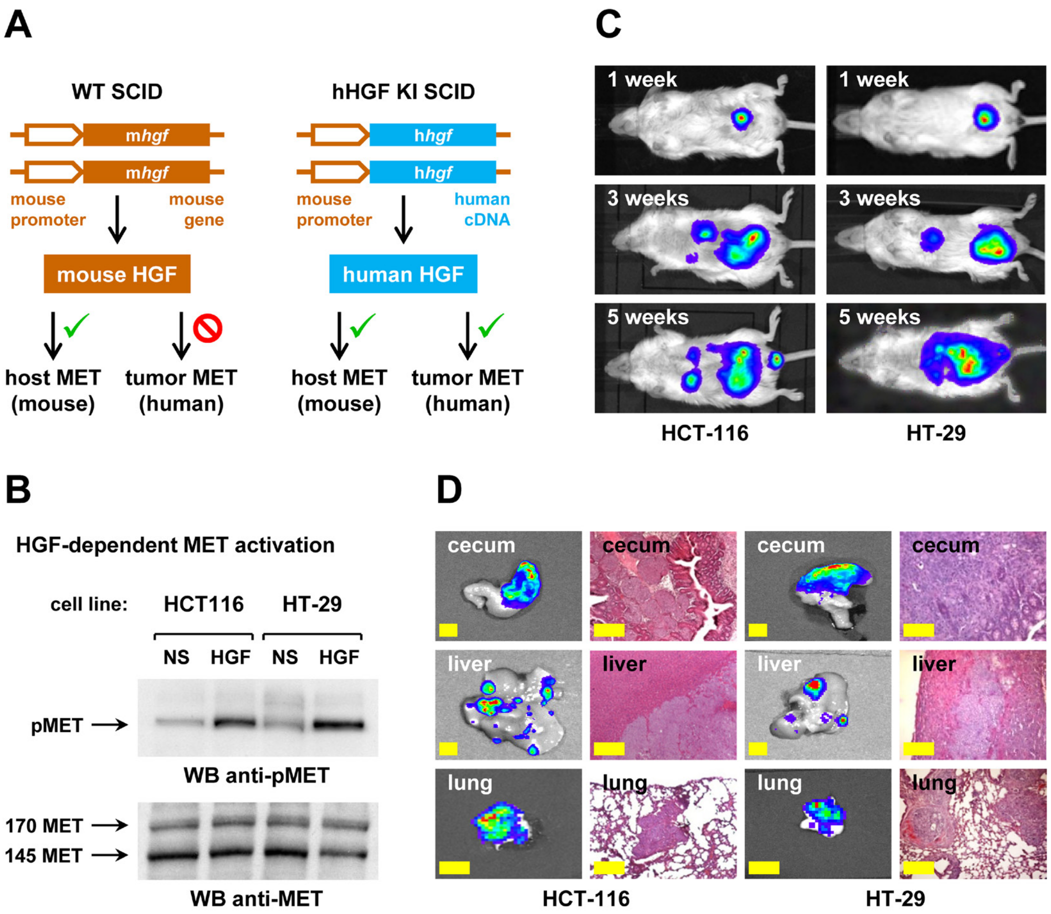

Supplementary Figure S1: Set up of an orthotopic mouse model of metastatic colorectal cancer that reproduces species-specific paracrine HGF signaling. (A) Schematic representation of the wild-type (WT) and human HGF knock-in (hHGF KI) SCID mouse strains. (B) HCT-116 (G13D KRAS) and HT-29 (V600E BRAF) human CRC cells display HGF-dependent MET activation. NS, not stimulated; WB, Western blot. The 170 kDa band corresponds to the non-mature MET precursor. (C) HCT-116 or HT-29 cells engineered to express luciferase were micro-injected into the cecum submucosa of hHGF KI SCID mice. Tumor engraftment and development was followed over time by in vivo bioluminescence imaging. (D) Orthotopic tumors disseminate following the same metastatic pattern observed in CRC patients. Metastatic dissemination to the liver and lung can be conveniently detected at autopsy by ex vivo bioluminescence analysis of explanted organs. Bar size for bioluminescence images: 5 mm. Bar size for histological images: 250 µm.

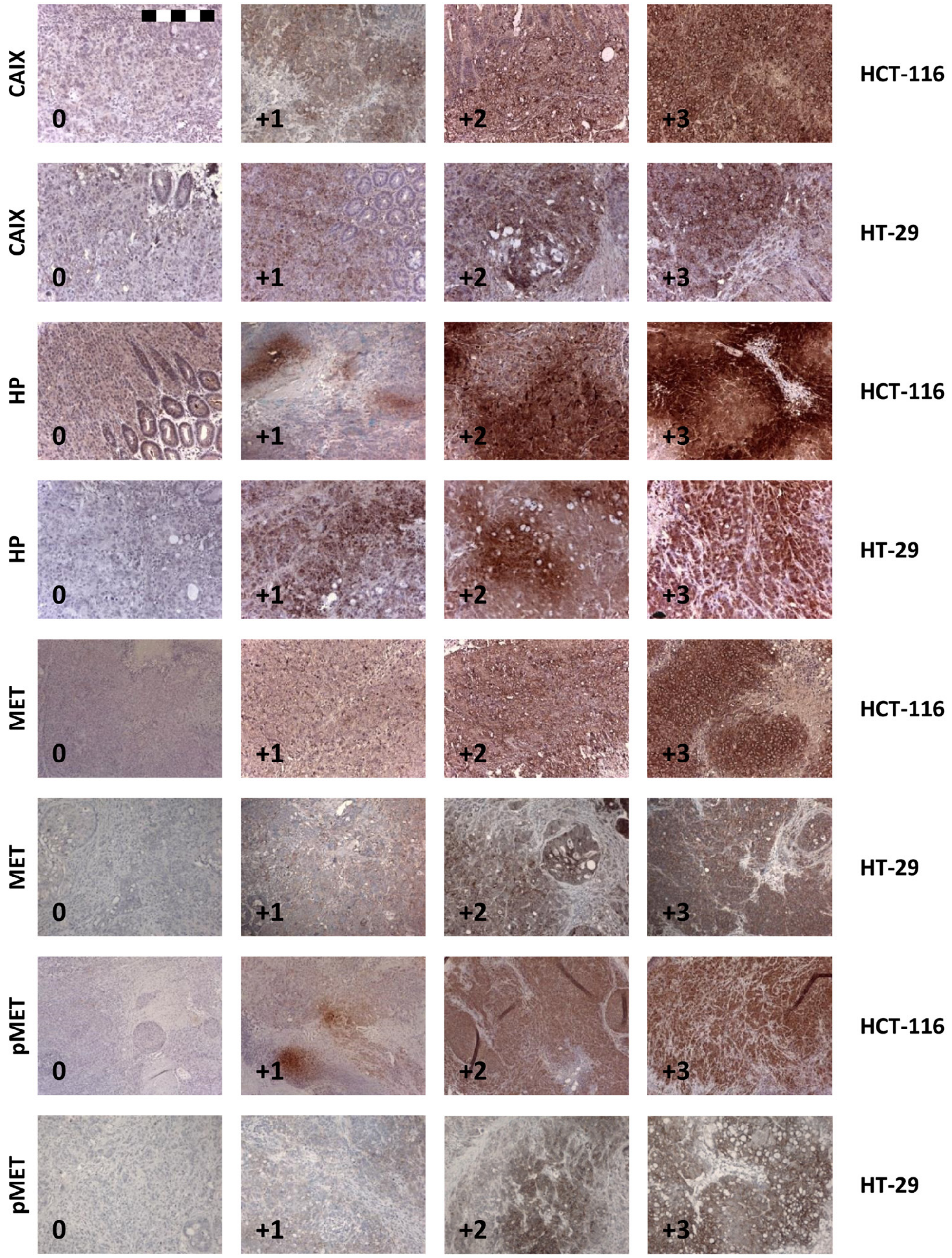

Supplementary Figure S2: Immunohistochemistry scoring system. To assess tumor hypoxia and MET expression/activation, HCT-116 or HT-29 tumor sections were processed for immuno-histological analysis and then stained with anti-carbonic anhydrase IX antibodies (CAIX), hypoxiprobe antibodies (HP), anti-total MET antibodies (MET), or anti-phospho-MET antibodies (pMET). Stained sections were analyzed by microscopy and confronted with the reference images shown above. Using this scale system, each tumor was given a score, and an average score was calculated for each experimental group (see Figures 1 and 2). Bar size: 500 µm.

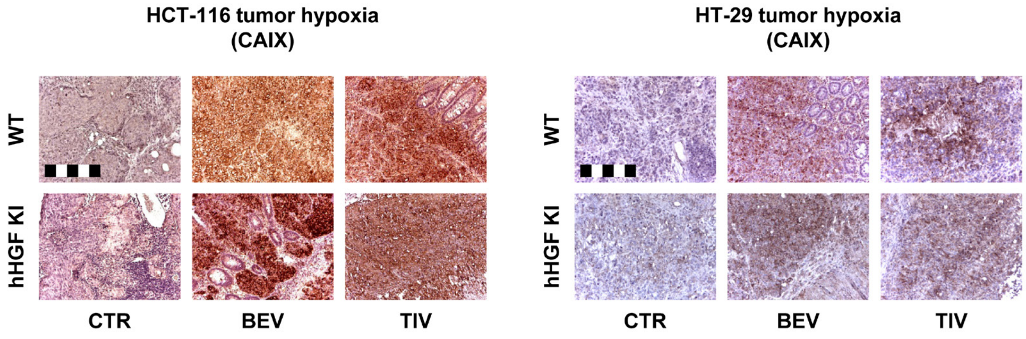

Supplementary Figure S3: Anti-angiogenic therapy promotes tumor hypoxia of orthotopic colorectal tumors. HCT-116-luc and HT-29-luc cells were orthotopically injected into wild-type (WT) or human HGF knock-in (hHGF KI) SCID mice, and tumor-bearing animals were randomly assigned to 3 treatment arms: control (CTR); 15 mg/kg bevacizumab (BEV); 10 mg/kg tivozanib (TIV). After 4 weeks of treatment, mice were sacrificed and tumors extracted for analysis. Tumor hypoxia was determined by IHC analysis of tumor sections stained with anti-carbonic anhydrase IX (CAIX) antibodies. Bar size: 250 µm. See also Figure 2A for quantitative data.

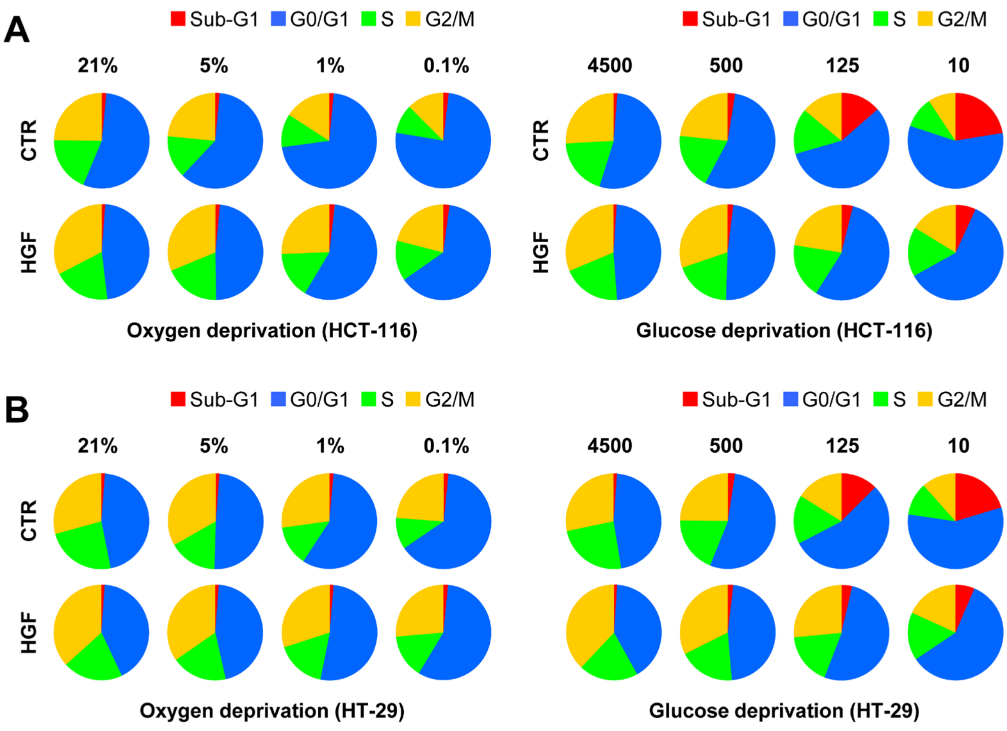

Supplementary Figure S4: Oxygen and glucose deprivation differentially affects cell cycle progression of colorectal cancer cells. (A) HCT-116 or HT-29 cells were incubated in progressively lower oxygen concentrations (21-0.1%) in the absence or presence of 50 ng/ml HGF, and cell cycle distribution was determined by DNA content analysis. (B) HCT-116 or HT-29 cells were incubated in progressively lower glucose concentrations (4500-10 mg/L) in the absence or presence of 50 ng/ml HGF, and cell cycle distribution was determined as above. Values represent the mean of 3 independent experiments.

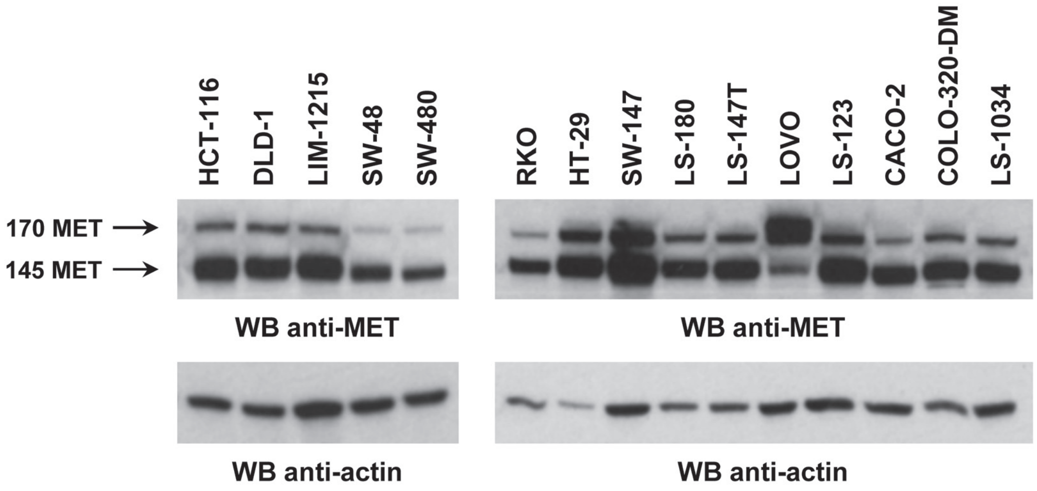

Supplementary Figure S5: Western blot analysis of MET expression in a panel of human colorectal cancer cells. The indicated human colorectal cancer cells were lysed and then analyzed by Western blotting (WB) using anti-human MET antibodies. The same blots were also analyzed by Western blotting with anti-human actin antibodies. The 170 kDa band corresponds to the non-mature MET precursor.
